# A genome-wide analysis of the lysophosphatidate acyltransferase (LPAAT) gene family in cotton: organization, expression, sequence variation, and association with seed oil content and fiber quality

**DOI:** 10.1186/s12864-017-3594-9

**Published:** 2017-03-01

**Authors:** Nuohan Wang, Jianjiang Ma, Wenfeng Pei, Man Wu, Haijing Li, Xingli Li, Shuxun Yu, Jinfa Zhang, Jiwen Yu

**Affiliations:** 1 National Key Laboratory of Cotton Biology, Institute of Cotton Research, Chinese Academy of Agricultural Sciences, Anyang, 455000 China; 2 College of Agronomy, Northwest A&F University, Yangling, 712100 China; 3 Department of Plant and Environmental Sciences, New Mexico State University, Las Cruces, 880033 USA

**Keywords:** *Gossypium* spp, Lysophosphatidic acid acyltransferase (LPAAT), Gene expression patterns, Sequence variation, Seed oil

## Abstract

**Background:**

Lysophosphatidic acid acyltransferase (LPAAT) encoded by a multigene family is a rate-limiting enzyme in the Kennedy pathway in higher plants. Cotton is the most important natural fiber crop and one of the most important oilseed crops. However, little is known on genes coding for LPAATs involved in oil biosynthesis with regard to its genome organization, diversity, expression, natural genetic variation, and association with fiber development and oil content in cotton.

**Results:**

In this study, a comprehensive genome-wide analysis in four *Gossypium* species with genome sequences, i.e*.*, tetraploid *G. hirsutum*- AD_1_ and *G. barbadense*- AD_2_ and its possible ancestral diploids *G. raimondii*- D_5_ and *G. arboreum*- A_2_, identified 13, 10, 8, and 9 *LPAAT* genes, respectively, that were divided into four subfamilies. RNA-seq analyses of the *LPAAT* genes in the widely grown *G. hirsutum* suggest their differential expression at the transcriptional level in developing cottonseeds and fibers. Although 10 *LPAAT* genes were co-localised with quantitative trait loci (QTL) for cottonseed oil or protein content within a 25-cM region, only one single strand conformation polymorphic (SSCP) marker developed from a synonymous single nucleotide polymorphism (SNP) of the *At-Gh13LPAAT5* gene was significantly correlated with cottonseed oil and protein contents in one of the three field tests. Moreover, transformed yeasts using the *At-Gh13LPAAT5* gene with the two sequences for the SNP led to similar results, i.e., a 25–31% increase in palmitic acid and oleic acid, and a 16–29% increase in total triacylglycerol (TAG).

**Conclusions:**

The results in this study demonstrated that the natural variation in the *LPAAT* genes to improving cottonseed oil content and fiber quality is limited; therefore, traditional cross breeding should not expect much progress in improving cottonseed oil content or fiber quality through a marker-assisted selection for the *LPAAT* genes. However, enhancing the expression of one of the *LPAAT* genes such as *At-Gh13LPAAT5* can significantly increase the production of total TAG and other fatty acids, providing an incentive for further studies into the use of *LPAAT* genes to increase cottonseed oil content through biotechnology.

**Electronic supplementary material:**

The online version of this article (doi:10.1186/s12864-017-3594-9) contains supplementary material, which is available to authorized users.

## Background

Cotton (*Gossypium* spp.) is not only the world's most important fiber crop, but also an irreplaceable oil crop. The cotton fiber provides 85% of the farm gate value of the cotton crop, and the rest is made up by cottonseed, seed meal and seed oil (National Cottonseed Association, www.cottonseed.org). Cottonseed oil represents a complementary product that can be used in foods or as a material for biodiesel production, with quality and price advantages over rapeseed oil and soybean oil. Cottonseed oil, which makes up approximately 16% of the seed weight, is the most valuable product derived from cottonseed [1]. As a result, there has been a stable increase in the demand for cottonseed oil in the global market. At present, there are two main strategies to genetically improve cottonseed oil yields, i.e., traditional breeding to increase the proportion of oil in cottonseed and transgenic approaches to increase the quality and content of cottonseed oil. However, it is currently unknown if an improvement in cottonseed oil production can be achieved through manipulations of genes involved in plant oil biosynthesis.

Plant oils are mainly composed of triacylglycerols (TAGs), the main storage lipids. There are two pathways for TAG biosynthesis in plants [2]. The first pathway is the *de novo* biosynthesis from glycerol-3-phosphate and acyl-CoA occurs via the Kennedy pathway, which involves three acyltransferases, i.e., glycerol-3-phosphate O-acyltransferase (GPAT), lysophosphatidic acid acyltransferase (LPAAT), and diacylglycerol acyltransferase (DGAT) [2]. In mammals, LPAATs are identified as part of the 1-acyl-glycerol-3-phosphate O -acyltransferase (AGPAT) family with LPAAT 1 to 5 corresponding to AGPAT 1 to 5, and LPAAT ƞ to AGPAT 7 [3, 4]. DGAT belongs to the membrane-bound O-acyltransferase (MBOAT) family that includes four subfamilies in plants, i.e., MBOAT1, DGAT1, lysophosphatidylcholine acyltransferase (LPCAT) or lysophospholipid acyltransferase (LPLAT), and homologs of *Saccharomyces cerevisiae* glycerol uptake protein (GUP) [5–7]. GPAT, AGPAT, LPCAT or MBOAT share common features in acyltransferase motifs, but their overall sequences are distinctly different [3]. The second pathway involves the conversion of lipid phosphatidylcholine (PC) to diacylglycerol (DAG), in which, acyl-CoAs are redirected from PC via the activities of phospholipase C, choline phosphotransferase, and phosphatidylcholine:diacylglycerol cholinephosphotransferase (PDCT) [8, 9], or phospholipid:diacylglycerol acyltransferase (PDAT) [10]. An acyl group can be released from PC to generate lysophosphatidylcholine (LPC) by the reverse reaction of acyl-CoA:LPC acyltransferase [11] or by phospholipase A/acyl-CoA synthase [12].

Phosphatidic acids (PAs) are a key intermediate in the biosynthesis of TAGs and the first acylation step catalyzed by GPAT can be partly bypassed by dihydroxyacetone phosphate acyltransferase in some tissues and organisms. Therefore, LPAATs are vital to PA biosynthesis in catalyzing the incorporation of acyl groups into the *sn*-2 position of the glycerol backbone [13]. In higher plants, two pathways of LPAAT catalysis control the metabolic flow of lysophosphatidate (LPA) into different PAs in diverse tissues. PAs are either dephosphorylated to form TAGs, or are used in the synthesis of phospholipids, which are important components of biological membranes. Therefore, LPAATs are crucial to the biosynthesis of membrane phospholipids and storage lipids in developing seeds. In fact, in higher plants, LPAATs play an essential role in improving the fatty acid component of seeds by reducing the proportion of saturated fatty acids (SFAs).

LPAATs are thought to be among the most stringent acyltransferases in terms of substrate specificity [14–17]. Studies on the acyl specificity of LPAATs in various plant species have shown that they have stronger affinity for 16–18 carbon monounsaturated fatty acids (MUFAs) than for SFAs [18]. There are at least two classes of *LPAAT* genes (classes A and B) in different plant species [14]. The characteristics of class A microsomal *LPAATs*, as defined by Frentzen [14], are ubiquitously expressed throughout the plant, and their enzymes show specificity for 18:1-CoA. These are typical features of enzymes that biosynthesize membrane glycerolipids. Class B *LPAATs* were first cloned and characterized from coconut (*Cocos nucifer*) [19]. In this subfamily, *LPAAT* genes are generally expressed in seeds, and they encode enzymes with a strong substrate specificity towards unusual acyls, such as erucic acid (22:1) and lauric acid (12:0), consistent with the seed microsomal activities and oil composition of the above species. However, recent studies have indicated that the characteristics of B-class LPAATs may differ in other species. For example, the B-class LPAATs in castor (*Ricinus communi*) showed specificity for 18:1-OH [20].

In a recent study, a rapeseed (*Brassica napus*) LPAAT was shown to be the rate-limiting enzyme catalyzing the conversion of lysophosphatidate (LPA) to PA in the TAG assembly process [21]. The expression of rapeseed *LPAAT* genes in *Arabidopsis* enhanced LPAAT enzymatic activities, which redirected more acyl-CoAs from PC and relieved feedback inhibition of TAG biosynthesis. Also, the overexpression of *LPAATs* increased seed oil content, thus reinforcing the utility of *LPAAT* genes as valuable biotechnological tools. Consequently, *LPAAT* homologs have been cloned from poached eggplant (*Limnanthes douglasii*) [22], *A. thaliana* [23], rapeseed [24], coconut [19], corn (*Zea mays*) [25], Indian cress (*Tropaeolum majus*) [26], Java olives (*Sterculia foetida*) [27], and castor [20]. The overexpression of a *LPAAT* gene from the *SLCl-1* mutant yeast stain in *A. thaliana* and *B. napus* increased the incorporation of long-chain fatty acids into the *sn*-2 position of TAGs, leading to an increase in seed oil content by 8–48% [28]. However, thus far, no such a transgenic work has been performed using *LPAAT* genes from cotton.

To date, little is known about the natural variation of the *LPAAT* genes and their association with seed oil production in higher plants including cotton. Furthermore, none of the genes encoding for these enzymes has been characterized in cotton; therefore, their role in increasing cottonseed oil content is unknown. In addition, since fatty acids are essential for membrane biosynthesis, saturated very-long-chain fatty acids were found to promote fiber cell elongation in cotton [29]. However, the relationship between natural genetic variation in *LPAAT* genes and fiber development is also currently unknown. Near-isogenic lines (NILs) or backcross inbred lines (BILs) with the same genetic background but differing in oil content, fiber initiation or elongation can provide important genetic stocks to investigate *LPAAT* genes with relation to fiber development and seed oil content.

The genomic organization of the *LPAAT* family has been mostly studied in only *Arabidopsis* and castor, but not in other species including cotton. Upland cotton (*G. hirsutum*-AD_1_) with higher yields and wider adaptations produces approximately 95% of the world cotton fibers and seed. Another cultivated tetraploid, i.e., *G. barbadense-*AD_2_, accounting for the remaining world cotton production, produces much longer, stronger and finer fibers with higher cottonseed oil content and lower protein content than Upland cotton. Therefore, BILs developed from an interspecific crossing between the two species provide important genetic stocks to address if *LPAATs* are associated with cottonseed oil content and fiber quality. The genomes of the two cultivated tetraploid cotton species were recently sequenced [30–33]. It is commonly accepted that, like other three wild tetraploid cotton species including *G. tomentosum*-AD_3_ *, G. mustelinum*-AD_4_, and *G. darwinii*-AD_5_, both cultivated tetraploid species originated from a common ancestor of a natural hybrid between an extant diploid A-genome cotton (likely tree cotton *G. arboreum-*A_2_ or levant *G. herbaceum-*A_1_) and a wild D genome (likely *G. raimondii-*D_5_) species [34]. In this study, we identified 40 *LPAAT* genes in the two allotetraploid species, i.e., *G. hirsutum* and *G. barbadense* and their likely ancestral diploid species *G. arboreum* and *G. raimondii* whose genomes were sequenced earlier [35–37]. After an in-silico analysis of the phylogeny, genomic organization and gene structure for the *LPAAT* gene family, we then conducted a detailed analysis of transcription profiles of the *LPAAT* gene family by analyzing information in two RNA-seq datasets followed by a quantitative real-time RT-PCR (qRT-PCR) analysis. Sequence variations within each gene were further evaluated for development of polymorphic markers and association analysis with cottonseed oil content and fiber quality traits. One of the *LPAAT* genes with a sequence variation was used in a transgenic yeast study to confirm its effect on TAGs. The study represents one of the most comprehensive genomic approaches in plants that combine an in-silico bioinformatics analysis with transcriptomics at the RNA level, SNP identification and typing at the DNA level and their associations with seed oil and fiber quality.

## Results

### Genome-wide identification of *LPAAT* genes and phylogenetic analysis

**Fig. 1:**
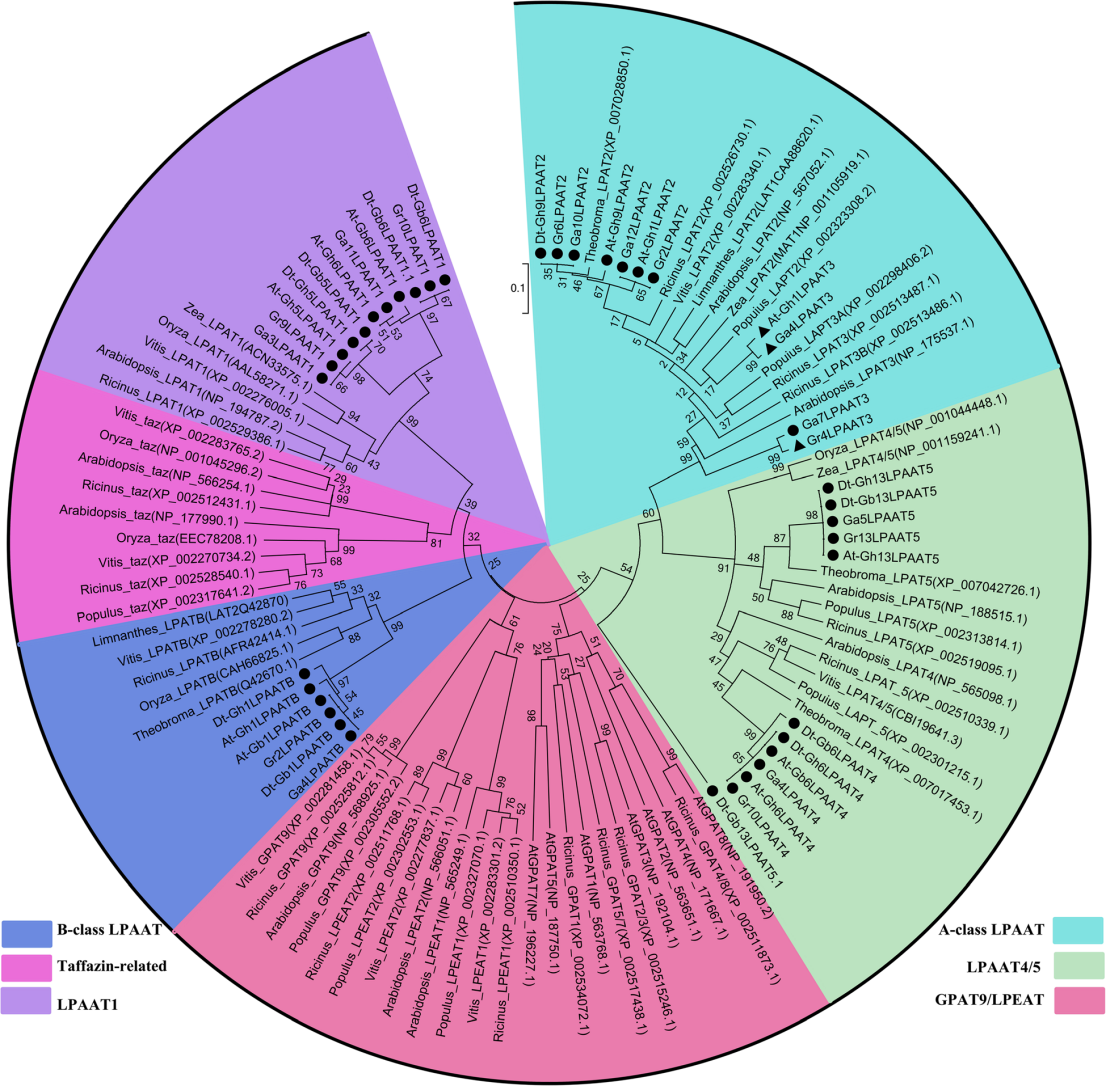
A phylogenetic tree of *LPAATs* in four *Gossypium* species. Sequences of other *LPAAT* genes and *LPAAT*-related genes were downloaded from NCBI website. ● *Gossypium*; ▼ three putative isoforms in *Gossypium*

The whole genome sequence scaffolds from the ancestral diploids *G. raimondii* (D_5_), *G. arboreum* (A_2_) and their descendant tetraploids *G. hirsutum* (AD_1_) and *G. barbadense* (AD_2_) were used for a genome-wide search for *LPAAT* genes in *Gossypium*. As a result, we identified 40 *LPAATs* genes in the four genomes, including 8 *Gr_LPAATs* based on the sequence information of D_5_ reported by Paterson et al. [35], 9 *Ga_LPAATs* in the draft A_2_ genome reported by Li et al. [37], and 13 and 10 *LPAATs* on the A subgenome (*At-Gh* (*Gb*)*_LPAATs*) or the D subgenome (*Dt-Gh* (*Gb*)_*LPAATs*) in the draft AD1 genome [31] and AD2 genome [33], respectively. The further phylogenetic analysis of putative cotton LPAAT proteins included some previously characterized plant acyltransferases (Fig. 1). Based on their predicted protein sequences, the *LPAAT* genes were classified into four subfamilies; A-class LPAAT (LPAAT2/3) (11 genes), LPAAT4/5 (12 genes), B-class LPAAT (6 genes), and LPAAT1 (plastidial) (11 genes) (Table 1). Also, some close paralogous relatives of *LPAATs* were identified in the four *Gossypium* species.

**Table 1:** Characteristics of *LPAAT* genes and predicted properties of LPAAT proteins in four *Gossypium* species

Gene name	Gene identifier	Size (AA)	MW (KD)	*pI*	Subcellular localization
A-class LPAAT(LPAAT2/3) (Box I: NHXXDXD; Box III: FVEGTR)					
*Dt-Gh9LPAAT2*	Gh_D09G1169	387	43.5451	9.46	Endoplasmic reticulum
*Gr6LPAAT2*	Gorai.006G138600.1	387	43.501	9.53	Endoplasmic reticulum
*Ga10LPAAT2*	Cotton_A_07803	387	43.501	9.53	Endoplasmic reticulum
*At-Gh9LPAAT2*	Gh_A09G1164	387	43.492	9.63	Endoplasmic reticulum
*At-Gh1LPAAT2*	Gh_A01G1672	381	42.9697	9.75	Endoplasmic reticulum
*Ga12LPAAT2*	Cotton_A_15334	381	42.9297	9.75	Endoplasmic reticulum
*Gr2LPAAT2*	Gorai.002G230000.1	381	42.9136	9.68	Endoplasmic reticulum
*At-Gh1LPAAT3*	Gh_A01G1107	230	25.8612	9.65	Plasma membrane
*Ga4LPAAT3*	Cotton_A_39902	260	29.2383	8.95	Plasma membrane
*Ga7LPAAT3*	Cotton_A_12015	370	42.0493	9.11	Endoplasmic reticulum
*Gr4LPAAT3*	Gorai.004G019300.1	204	22.9039	9.4	Mitochondrial inner membrane
LPAAT4/5 LPAAT (Box I: NHXXDXD; Box III: FVEGTR)					
*Dt-Gh6LPAAT4*	Gh_D06G0073	377	43.6875	8.86	Plasma membrane
*Gr10LPAAT4*	Gorai.010G012300.1	377	43.6663	8.77	Plasma membrane
*At-Gh6LPAAT4*	Gh_A06G0100	377	43.7905	8.77	Plasma membrane
*Ga4LPAAT4*	Cotton_A_06880	377	43.7715	8.67	Plasma membrane
*Dt-Gb6LPAAT4*	Gbscaffold17837.25.0	377	43.7625	8.77	Plasma membrane
*At-Gb6LPAAT4*	Gbscaffold22202.16.0	377	43.6603	8.77	Plasma membrane
*Gr13LPAAT5*	Gorai.013G052400.1	379	44.103	8.59	Plasma membrane
*At-Gh13LPAAT5*	Gh_A13G0409	354	41.1643	8.9	Mitochondrial inner membrane
*Dt-Gh13LPAAT5*	Gh_D13G0458	372	432.95	8.79	Plasma membrane
*Ga5LPAAT5*	Cotton_A_17180	384	44.6355	9.06	Plasma membrane
*Dt-Gb13LPAAT5.1*	Gbscaffold19187.1.0	255	29.6291	9.14	Plasma membrane
*Dt-Gb13LPAAT5*	Gbscaffold19187.1.3	372	43.332	8.9	Plasma membrane
B-class LPAAT (Box I: NHXSXXD; Box III: FPEGT)					
*At-Gh1LPAATB*	Gh_A01G0616	358	40.2893	9.69	Endoplasmic reticulum
*Dt-Gh1LPAATB*	Gh_D01G0632	311	35.1431	9.78	Mitochondrial inner membrane
*Ga4LPAATB*	Cotton_A_04784	288	32.8554	9.65	Mitochondrial inner membrane
*Gr2LPAATB*	Gorai.002G088200.1	299	33.7234	9.79	Mitochondrial inner membrane
*Dt-Gb1LPAATB*	Gbscaffold8424.3.0	311	35.1891	9.75	plasma membrane
*At-Gb1LPAATB*	Gbscaffold10133.29	311	35.2282	9.77	plasma membrane
LPAAT1 (Plastidial) (Box I: NHQXXXD; Box III: FPEGT)					
*Ga3LPAAT1*	Cotton_A_01233	312	34.2761	9.8	Microbody (peroxisome)
*At-Gh5LPAAT1*	Gh_A05G0428	358	39.6192	9.8	Plasma membrane
*Gr9LPAAT1*	Gorai.009G056600.1	355	39.3199	9.72	Plasma membrane
*Dt-Gh5LPAAT1*	Gh_D05G0546	355	39.392	9.69	Mitochondrial inner membrane
*Gr10LPAAT1*	Gorai.010G113400.1	335	37.306	9.81	Plasma membrane
*Ga11LPAAT1*	Cotton_A_25757	238	26.2541	9.65	Plasma membrane
*Dt-Gb5LPAAT1*	Gbscaffold12734.5.1	319	35.4066	9.59	Microbody (peroxisome)
*Dt-Gb6LPAAT1*	Gbscaffold3314.7.0	230	25.4521	9.65	Endoplasmic reticulum
*At-Gb6LPAAT1*	Gbscaffold6506.1.0	322	35.6169	9.8	Plasma membrane
*Dt-Gb6LPAAT1.1*	Gbscaffold3314.8.0	335	37.308	9.74	Endoplasmic reticulum
*At-Gh6LPAAT1*	Gh_A06G0884	322	35.7141	9.85	Golgi body

*AA* amino acid, *pI* theoretical isoelectric point, *MW* theoretical molecular weight, *KD* Kilo Dalton

The LPAAT2/3 subfamily, also known as the A-class LPAAT family, encodes microsomal LPAATs that show a generalized expression pattern. This subfamily includes *AtLPAAT2* from *Arabidopsis* [23]. Within the LPAAT2/3 subfamily, *Gr6LPAAT2, Ga10LPAAT2* and two copies of *GhLPAAT2* from tetraploid *G. hirsutum* (*Dt-Gh9LPAAT2* and *At-Gh9LPAAT2*) encoded proteins with a high level of similarity to the cacao TcLPAAT2 protein, and were identified as putative orthologs from different genomes or subgenomes, i.e., homoelogs (Fig. 1). Interestingly, three putative LPAAT2/3 isoforms were identified in *Gossypium*. These three LPAAT isoforms appeared to have incomplete conserved domains (Additional file 1: Figure S2). Specifically, *Ga4LPAAT3* and *At-Gh1LPAAT3* had only one LPAAT-characteristic Box III (ΦFPEGTR-G, where Φ is a hydrophobic residue) [38], a motif that is well conserved among proteins with the ΦFVEGTR consensus sequence in the LPAAT2/3 subfamily. Gr4LPAAT3 had Boxes III and IV (ΦPΦΦPΦΦΦ), but an imprecise VLIPRTKG consensus sequence. The sequence around Box I (Φ-NHQS-ΦDΦΦ) was quite similar among the LPAAT2/3 proteins, conforming to the consensus sequence NHXSDIDWL, where X usually indicates an arginine (R) residue [38]. However, this consensus sequence was different (NHVSDSDTΦ) in *Gr7LPAAT3*. The same held true for sequences around Box II (G-ΦFIDR), where there was a well-conserved E(D)YLFLER motif in most members of the cotton LPAAT2/3 subfamily (Additional file 1: Figure S2), with an exception of *Gr7LPAAT3* having a different sequence (ESIFLDR). The consensus sequences of Boxes III and IV were conserved among all of the LPAAT2/3 proteins.

Twelve *LPAATs* were clustered into the LPAAT4/5 subfamily which was a closely related sister group with LPAAT2/3 (Fig. 1), although there were some sequence differences around various LPAAT boxes. For example, there was an aspartic acid (D) residue near Box I in the LPAAT2/3 subfamily, but a glutamic acid (E) residue near Box I in the LPAAT4/5 subfamily. This variation may explain why these sequences were grouped into two subfamilies.

Six *Gossypium* gene sequences designated as a paralogous *LPAAT* group clustered together in the B-class LPAAT subfamily, which encodes LPAATs that are usually, but not always, seed-specific and shows substrate specificities for unusual acyl groups [14]. As the sister group to the B-class subfamily the LPAAT1 (plastidial) subfamily contained eleven *LPAATs* in two paralogous groups from two different branches. The LPAAT1 (plastidial) subfamily includes *AtLPAAT1* in *Arabidopsis* [39] (Fig. 1). A close relationship between B-class LPAATs and plastidial LPAATs was revealed in the phylogenetic analysis, consistent with the results of a previous report [39]. The B-class LPAATs and the plastidial LPAATs shared the same conserved motif (FPEGT) around Box III, except for Ga4LPAATB which lacked Box III (Additional file 2: Figure S3).

In plants and other organisms, LPAAT activities have been detected in the endoplasmic reticulum [23], plasma membrane [39], and mitochondria [40]. Table 1 summarizes their predicted subcellular localizations of the putative LPAAT proteins in cotton, together with information on the predicted length, molecular weight (MW), and isoelectric point (*pI*). Each LPAAT subfamily was predicted to a specific subcellular location. For example, within the A-class LPAAT (LPAAT2/3) subfamily, 72.7% of the LPAAT proteins were predicted to localize in the endoplasmic reticulum; and LPAAT4/5, B-class LPAATs, and LPAAT1 (plastidial) were predicted to be in the plasma membrane, the mitochondrial inner membrane, and the plasma membrane, respectively (Table 1).

### Structure and domain analysis of putative *LPAAT* genes in sequenced diploid and tetraploid *Gossypium* genomes

**Fig. 2:**
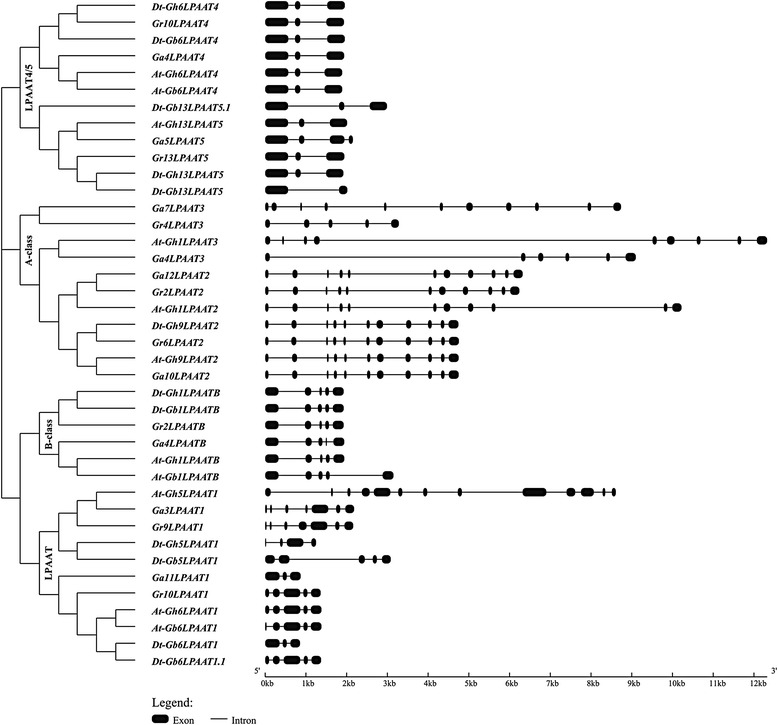
Genetic structures of *LPAAT* genes in four *Gossypium* species*. Black boxes* show exons and lines show introns

We used the GFF (Generic Feature Format) files of the four *Gossypium* species to analyze the exon–intron structure of putative *LPAAT* genes. Figure 2 shows the exon–intron structure of each subfamily. The number and location of introns varied among subfamilies, but there were some common features. In the A-class subfamily, eight deduced genes included 11 exons, but the locations of introns differed. *At-Gh1LPAAT3*, *Ga4LPAAT3* and *Gr4LPAAT3* were closely related to *LPAAT2*, but had only nine, six and five exons, respectively. Within this subfamily, homoelogous *LPAATs* had the same structure, such as *Dt-Gh9LPAAT2*, *Gr6LPAAT2*, *At-Gh9LPAAT2* and *Ga10LPAAT2*. Members of the LPAAT4/5 subfamily had three exons, except for *Ga5LPAAT5* and *Dt-Gb13LPAAT5* which had four and two exons, respectively. These similarities in gene structure were also observed among members of the B-class and LPAAT1 (plastidial) groups (Fig. 2). The results of the domain analysis indicated the presence of a highly conserved LPLAT_LCLAT1-like domain in the A-class and LPAAT4/5 LPAATs, and an LPLAT_AGPAT-like domain in the B-class and plastidial LPAATs (Additional file 3: Table S3). In conclusion, members belonging to the same subfamilies of the phylogenetic tree had a similar gene structure and conserved motif, consistent with the results of the phylogenetic analysis.

### Chromosomal distribution of *LPAAT* genes on *Gossypium* genomes

**Fig. 3:**
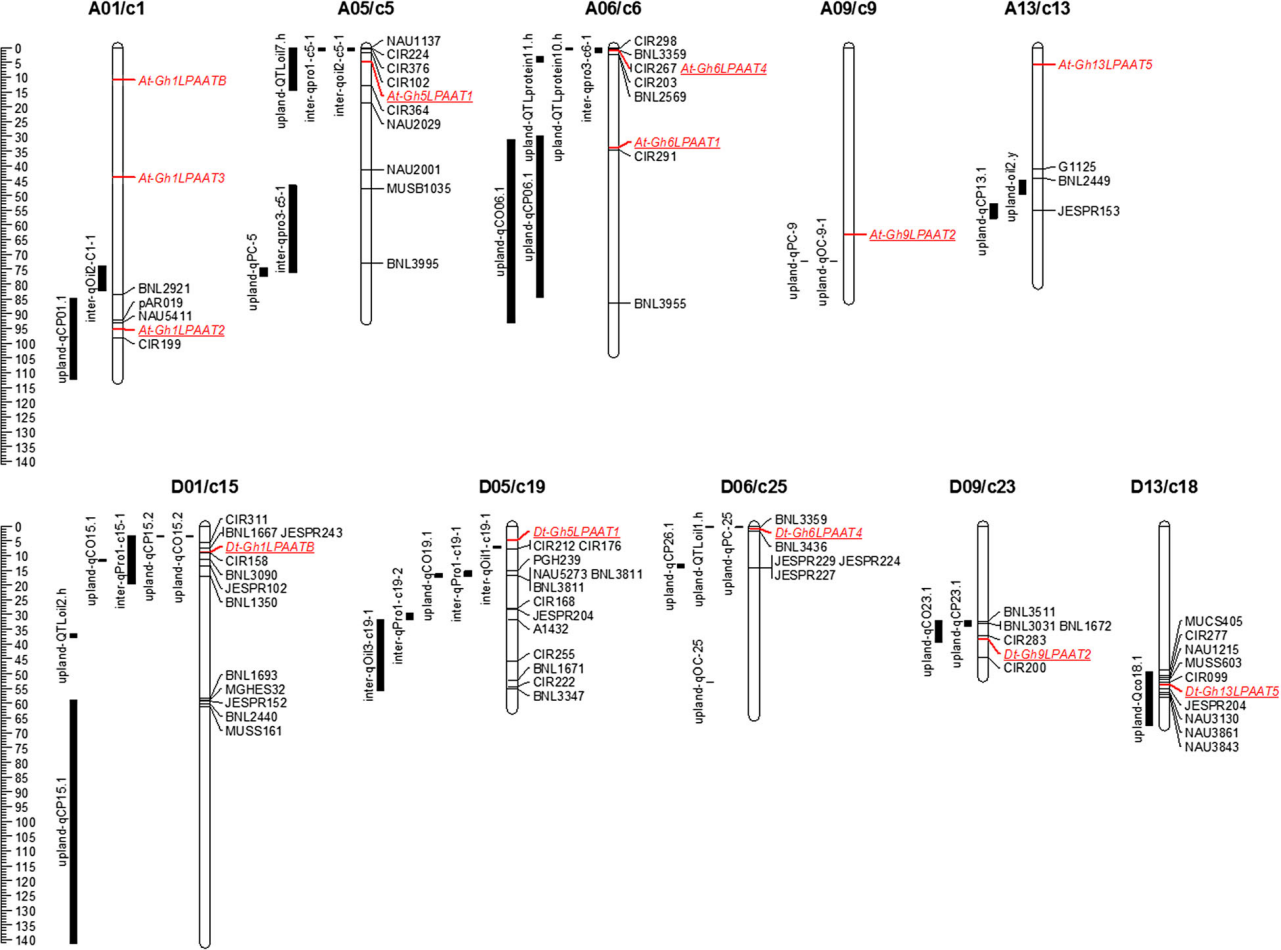
Co-localisation analysis of *LPAAT* genes with seed oil and protein quantitative trait loci (QTL). *Red color* shows *LPAAT* genes and *underlines* indicate the *LPAAT* genes co-localisation with seed oil and protein QTL

After integrating chromosomes of the sequenced cotton genomes excluding *G. barbadense* as its *LPAAT* genes were not physically well mapped, we found that the *LPAAT* family members were distributed unevenly among chromosomes. Among the candidate *LPAAT* genes, eight *GrLPAATs* were located in six D_5_ chromosomes (D_5_/c2, D_5_/c4, D_5_/c6, D_5_/c9, D_5_/c10, and D_5_/c13), and nine *GaLPAATs* to seven A_2_ chromosomes (A_2_/c3, A_2_/c4, A_2_/c5, A_2_/c7, A_2_/c10, A_2_/c11, and A_2_/c12) (Additional file 4: Figure S4). Because there are many syntenic gene blocks between the chromosomes of *G. raimondii* and *G. arboreum*, the dotted lines in Additional file 4: Figure S4 link homoelogous *LPAATs* between A_2_ and D_5_ chromosomes, but the chromosomes linked may not be homoelogous between genes from the two subgenomes of the tetraploid species. For *G. barbadense*, three *GbLPAATs* matched to the At subgenome, and seven *GbLPAATs* matched to the Dt subgenome (Additional file 4: Figure S4). For *G. hirsutum*, five *GhLPAATs* were on the Dt subgenome, and eight *GhLPAATs* on the At subgenome (Fig. 3).

### Analysis of transcript levels of *GhLPAAT* genes in two RNA-seq databases of tetraploid Upland cotton

**Fig. 4:**
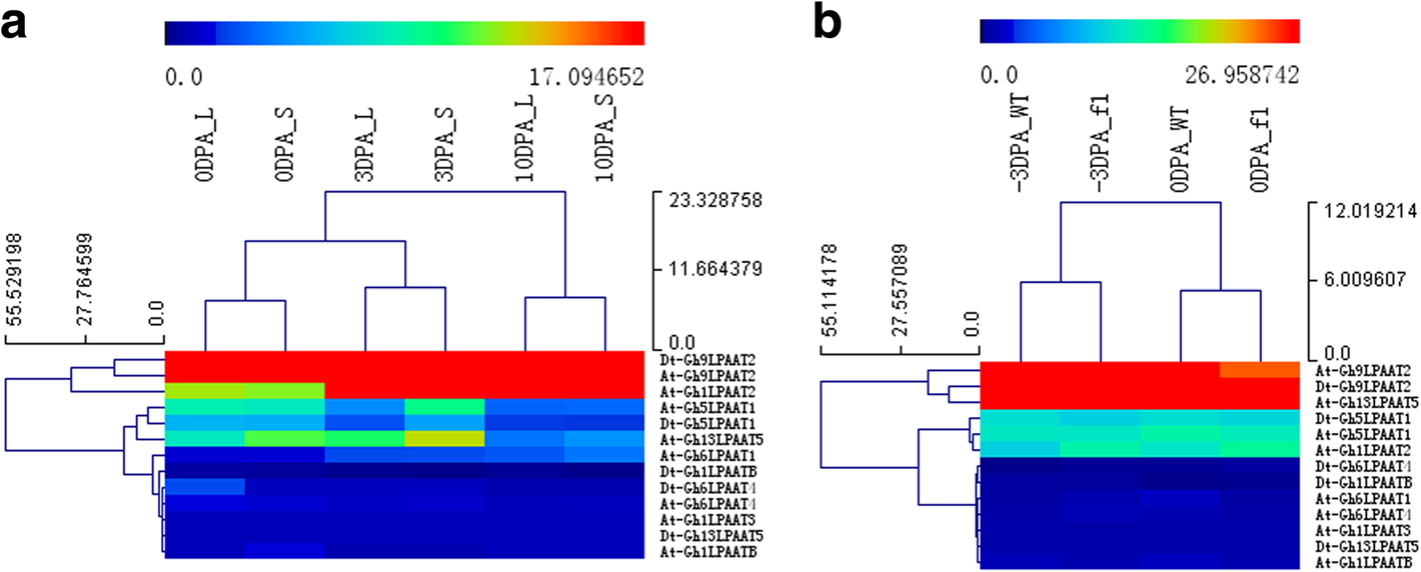
Transcript profiles of *LPAAT* genes in *Gossypium hirsutum*. **a** A heat map showing transcript levels of 13 *LPAAT* genes in ovules at three stages (0 DPA, 3 DPA, 10 DPA; shown above each lane) in two BILs. **b** Transcript levels of 13 *LPAAT* genes in −3 DPA and 0 DPA ovules of Xuzhou 142 and Xuzhou 142 *fl*. Color scale above dendrogram shows relative transcript levels. "L" and "S" indicate long fiber length line NMGA-062 and short fiber length line NMGA-105, respectively. "WT" and "*fl*" indicate Xuzhou 142 and its Xuzhou 142 *fl* mutant, respectively

To reveal a general pattern of gene expression for the *LPAAT* genes identified, we analyzed the transcript profiles of *LPAAT* genes in two RNA-seq datasets: one with transcriptomic information for two Upland BILs, i.e., NMGA-062 vs. NMGA-105 (with *G. barbadense* germplasm introgression), with differing fiber lengths but similar seed oil content, and the other containing transcriptomic information for Upland Xuzhou 142 vs. its fiberless and fuzzless mutant Xuzhou 142 *fl* (with a likely *G. barbadense* origin) [41] which differed in fiber initiation and cottonseed oil content [42]. The transcript abundances of all the 13 putative *LPAAT* genes were determined at the fiber initiation stage (0 DPA) and the elongation stage (3–10 DPA). Of the 13 genes in the sequenced *G. hirsutum* genome, two (*At-Gh1LPAAT3* and *Dt-Gh13LPAAT5*) was not expressed. The other 11 genes were transcribed at both stages of fiber development and in all tissues represented in the two RNA-seq datasets. Based on the RNA-seq data of the two BILs, the transcript levels of the A-class *LPAAT* genes were higher than those of the genes in the other three subfamilies in both genotypes. Interestingly, the transcript levels of *Dt-Gh9LPAAT2* and *At-Gh9LPAAT2* were similar in maintaining a high expression level at three different stages, but the *At-Gh1LPAAT2* had a low level of expression at 0 DPA fibers ovules, with the highest level observed in 10 DPA ovules in both genotypes (Fig. 4a). There was no significant difference in expression levels between the two BIL genotypes differing in fiber length but with similar seed oil content.

Similar patterns were observed in the other RNA-seq dataset for Xuzhou 142 and its fiberless and fuzzless mutant Xuzhou 142 *fl*. The exception was that *At-Gh13LPAAT5* showed higher transcript levels in Xuzhou 142 and Xuzhou 142 *fl* than in the other two BILs due to different genetic backgrounds (Fig. 4b). The results indicate that the expression of *LPAAT* genes at the transcription level is not involved in the natural variation of fiber length between Upland and *G. barbadense* and fiber initiation as controlled by the genes in Xuzhou 142 *fl*.

### Quantitative real-time RT-PCR analysis of transcript profiles of eight *GhLPAAT* genes

**Fig. 5:**
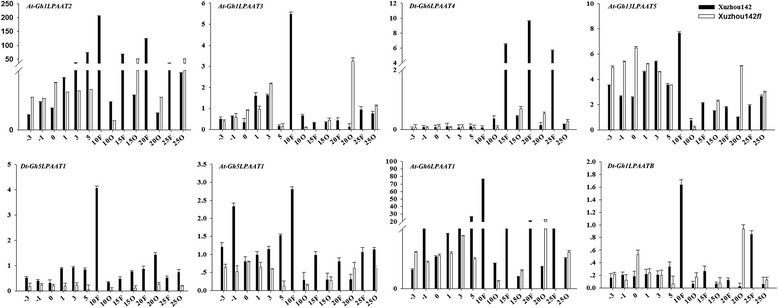
Expression patterns of eight *LPAAT* genes in Xuzhou 142 and Xuzhou 142 *fl*. Y-axis, relative expression levels. X-axis, days post anthesis. Fibers and ovules were not separated at −3, −1, 0, 1, 3, and 5 DPA. 10 F, 15 F, 20 F, 25 F DPA and 10O, 15O, 20O, and 25O DPA indicate fibers and ovules, respectively, at different stages. Error bars show standard deviation (S.D.) calculated from three replications

Due to a high homology for genes within the same subfamily, we chose eight of the above eleven expressed *LPAAT* genes in Upland cotton that have homologs with its ancestral diploid species *G. raimondii* and *G. arboreum* to design gene-specific primers (Additional file 3: Table S1) for a further evaluation of the transcript levels. The eight genes included two A-class genes, two *LPAAT4/5* genes, three plastidial *LPAAT* genes, and one B-class gene. Using qRT-PCR analyses, we further analyzed the gene transcript levels in roots, stems, leaves, petals, ovules, and fibers of Xuzhou 142 and the Xuzhou 142 *fl*. We analyzed ovules at different developmental stages, i.e., −3, −1, 0, 1, 3, and 5 DPA (fibers and ovules were not separated), and 10, 15, 20, and 25 DPA (fibers and ovules were separated) as cottonseed oil accumulates rapidly in ovules after 15–20 DPA. The results showed that the eight *LPAAT* genes were expressed in all the organs and tissues tested throughout the plant. However, seven *LPAAT* genes (except for *Dt-Gh6LPAAT4*) showed the highest transcript levels in 10 DPA fibers. *At-Gh6LPAAT1*, *At-Gh1LPAAT2*, *At-Gh1LPAAT3*, *At-Gh13LPAAT5* and *Dt-Gh1LPAATB* were expressed preferentially at the late period of ovule development in Xuzhou 142 *fl*, especially at 20 DPA. The transcript profiles of the two A-class genes were similar; however, in the LPAAT4/5 subfamily, *Dt-Gh6LPAAT4* and *At-Gh13LPAAT5* showed an opposite trend in their transcription patterns. At −3 to 5 DPA ovules (Xuzhou 142 and Xuzhou 142 *fl*) and 10 DPA fibers (Xuzhou 142), *Dt-Gh6LPAAT4* showed very low transcript levels, while *At-Gh13LPAAT5* was highly expressed (Fig. 5). Moreover, *Dt-Gh6LPAAT4* showed very high transcript levels, but *At-Gh13LPAAT5* had a low level of expression at 15–25 DPA fibers. In the B-class subfamily, *Dt-Gh1LPAATB* did not show a seed-specific expression pattern as expected, because it was also expressed in all of the other organs (Additional file 5: Figure S5).

### Co-localisation of *LPAAT* genes with quantitative trait loci (QTL) for seed oil and protein contents

To analyse if any of the *GhLPAATs* is genetically associated with cottonseed oil content, a co-localisation analysis of *GhLPAATs* with quantitative trait loci (QTL) for seed oil and protein contents was performed. We first downloaded QTL for these two traits from two types of genetic populations, i.e., intraspecific *G. hirsutum* and interspecific *G. hirsutum × G. barbadense* populations [43]. As a result, 10 *GhLPAATs* on five pairs of homeologous chromosomes were located within a 25 cM region of cottonseed oil or protein QTL, including *At-Gh1LPAAT2* on chromosome A01/c1, *At-Gh5LPAAT1* on A05/c5, *At-Gh6LPAAT4* and *At-Gh6LPAAT1* on A06/c6, *At-Gh9LPAAT2* on A09/c9, *Dt-Gh1LPAATB* on D01/c15, *Dt-Gh13LPAAT5* on D13/c18, *Dt-Gh5LPAAT1* on D05/c19, *Dt-Gh9LPAAT2* on D09/c23, and *Dt-Gh6LPAAT4* on D06/c25 except for *At-Gh13LPAAT5* on A13/c13 (Fig. 3). Interestingly, the respective cottonseed oil or protein QTL co-localised *LAPPT* genes on six chromosomes belong to three pairs of the homeologous chromosomes, i.e., *LPAAT1* on A05 and D05, *LPAAT2* on A09 and D09, and *LPAAT4* on A06 and D06, while other two pairs of homeologous chromosomes (i.e., A01 vs. D01 and A13 vs. D13) carry cottonseed oil or protein QTL but with different *LPAAT* genes. These co-localised QTL include five QTL (on A09 and D09, A13 and D13, and D06) from the Upland population only and five (on A01 and D01, A05 and D05, and A06) from both Upland and interspecific populations (Additional file 3: Table S4). However, since a 25-cM chromosomal region may contain several hundred genes [30, 31], the co-localisation of a seed oil QTL with a *LPAAT* gene may not indicate a causative relationship between the natural variation of the *LPAAT* gene and seed oil content (see next section).

### Sequence variation of *LPAAT* genes and its association with fiber quality and seed oil and protein contents

Sequence variations in the predicted genes between the sequenced *G. hirsutum* (TM-1) and *G. barbadense* (3–79 and Xinhai 21) were further analyzed. The results showed that most of the sequence variations were detected between homeologous genes from the A- and D- subgenomes, while only a few single nucleotide polymorphisms (SNPs) were predicted in homologous genes between the two cultivated species or between 3-79 and Xinhai 21 within *G. barbadense* (Additional file 6: Figure S6). The results imply that the differences in seed oil and protein contents and fiber quality traits between the two species are unlikely related to the natural sequence variations in the *LPAAT* genes, which is verified by the following correlation analysis between *LPAAT* gene markers and the seed and fiber quality traits.

The above RNA-seq datasets were further used to identify SNPs in the *LPAAT* genes within Upland cotton. In comparison with the sequenced TM-1 genome, we identified 43 SNPs in the cDNA sequences of seven *LPAAT* genes in the NMGA-062 and NMGA-105 dataset and 63 SNPs in five *LPAAT* genes in the Xuzhou 142 and Xuzhou 142 *fl* dataset (Additional file 3: Tables S5 and S6). However, as expected, most of the sequence differences were between TM-1 and the pair of the two BILs in the SG 747 background or the NILs in the Xuzhou 142 background (i.e., BILs vs. TM-1 or Xuzhou 142 WT/*fl* vs. TM-1). Only eight SNPs between NMGA-062 and NMGA-105 BILs were detected in five *LPAAT* genes (Additional file 3: Table S5). Between Xuzhou 142 and Xuzhou 142 *fl* NILs, eight SNPs were identified in four *LPAAT* genes, confirming the non-near-isogenic status of the two genotypes used in this study, which was recently reported by Ma et al. [41].

For an association analysis between the existence of SNPs in the *LPAAT* genes (detected from a comparison between the two BILs, i.e., NMGA-062 and NMGA-105) and cottonseed oil and protein contents and fiber traits, primers were designed to amplify fragments containing these SNPs using single strand conformation polymorphisms (SSCPs), which were used to screen the BIL population of 146 lines derived from a backcross between Upland SG 747 and *G. barbadense* Giza 75. As a result, one polymorphic SSCP marker was developed from a pair of primers (Additional file 3: Table S2) designed for *At-Gh13LPAAT5*, named At-Gh13LPAAT5-342 (Additional file 7: Figure S7). A transition, A/G (or T/C), was located in the 342th nucleotide of *At-Gh13LPAAT5* (Additional file 3: Table S6), but the mutation appears to be synonymous with no change in amino acid. To examine the relationship of the SSCP marker with seed oil and protein contents and fiber traits, a correlation analysis was performed in the BIL population of 146 lines tested in 2006, 2008 and 2009. The marker was found to be significantly associated with both seed oil and protein content in the BILs tested in 2008. The correlation between the presence of the SSCP marker and seed oil content was significantly negative (−0.281, *P* < 0.01) and significantly positive with seed protein content (0.245, *P* < 0.01) (Table 2). The results indicated that the presence of the SNP allele reduced cottonseed oil and increased protein content. Since seed oil and protein contents are negatively correlated (−0.905, *P* < 0.01), which was also reported by others [44], the opposite effects of the marker on seed oil and protein contents are expected. Moreover, the SSCP marker had a negative correlation with only one fiber quality trait, i.e., fiber uniformity (−0.176, *P* < 0.05) (Table 2). However, the above results obtained in 2008 were not confirmed by the results from the same BIL population tested in 2006 and 2009, as no significant correlations were detected between the SNP and seed oil and protein contents or fiber quality (Table 2). Therefore, the inconsistent association between the marker of this *LPAAT* gene and seed oil and protein contents or fiber quality indicates that the natural variation of this *LPAAT* gene may not affect seed oil and protein synthesis or fiber development, but more studies are needed. Overall, our results indicated that sequence variations in most if not all of the *LPAAT* genes are not associated with the natural variation (i.e., QTL) for seed oil content or fiber quality in cotton.

**Table 2:** The correlation coefficients between the marker At-Gh13LPAAT5-342 developed from the *LPAAT* gene and seed oil and protein contents and fiber quality traits in the backcross inbred line population of SG 747 × Giza 75 hybrid tested in 2006, 2008 and 2009

	2006	2008	2009
Fiber length (mm)	−0.082	−0.071	0.078
Fiber uniformity (%)	−0.063	−0.176^b^	0.02
Micronaire (unit)	−0.003	−0.089	0.091
Fiber strength (cN/tex)	−0.007	−0.042	0.088
Seed oil (%)	−0.122	−0.281^a^	0.119
Seed protein (%)	nt	0.245^a^	0.146

^a^ and ^b^ indicate correlation at the 0.01 and 0.05 significant levels, respectively. *nt* not tested

### Content of TAG and fatty acids in *At-Gh13LPAAT5* transgenic yeast strains

**Fig. 6:**
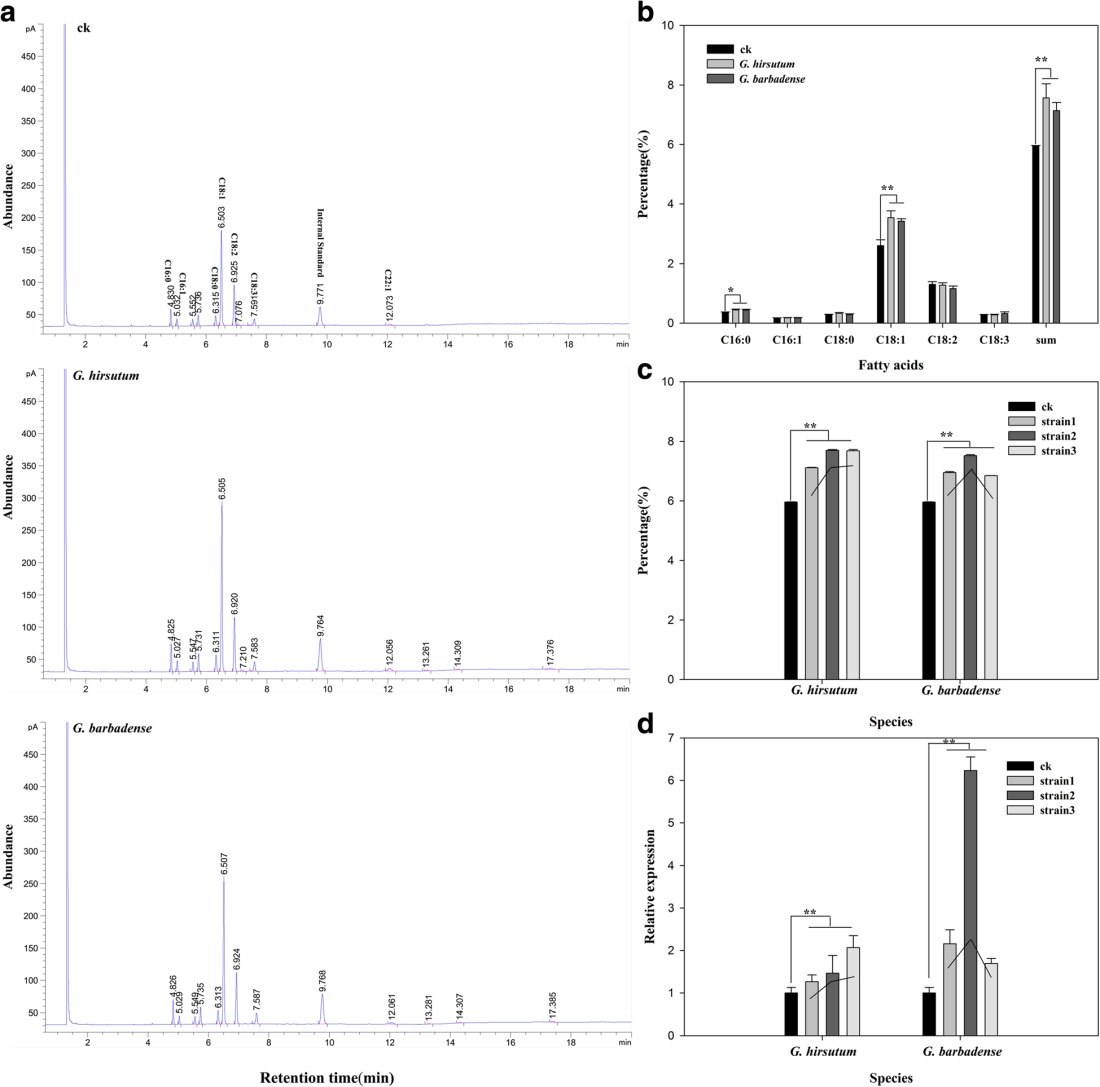
Content of TAG and fatty acids in *At-Gh13LPAAT5* transgenic yeast strains. **a** Gas chromatography/mass spectrometry chromatogram of fatty acids from transgenic yeasts. **b** The content of fatty acids in transgenic yeasts*.* **c** The total TAG content in three transgenic yeast strains from two backgrounds *G. hirsutum* and *G. barbadense*, respectively*.* **d** Expression patterns of *At-Gh13LPAAT5* in three transgenic yeast strains*.* ** and * indicate correlation at the 0.01 and 0.05 significant levels, respectively

Because *At-Gh13LPAAT5* is not located within the QTL region for the cottonseed oil and protein QTL on chromosome A13/c13 (Fig. 3) and the SNP detected in this gene was synonymous with no change in amino acid, the detected correlation between the SNP and cottonseed oil and protein content in the BIL population tested in 2008 should not be causative. To validate the hypothesis that both gene alleles producing polypeptides of the same sequence have the same effect on oil biosynthesis, we compared the effect of the *At-Gh13LPAAT5* gene from the Upland, *G. hirsutum* (as represented by sequences from TM-1 and Xuzhou 142) and *G. barbadense* (as represented by sequences from *G. barbadense* 3–79 and *G. hirsutum* Xuzhou 142 *fl*) sources on oil synthesis in transgenic yeasts. A full length cDNA with an 1119-bp open reading frame (ORF) from both sources was cloned into the yeast expression vector pPIC3.5 K and transformed into yeasts by LiCl. The GC/MS analysis determined six major fatty acids in the transgenic yeasts (represented by three transgenic yeasts for each source), i.e., palmitic acid (C16:0), hexadecenoic acid (C16:1), stearic acid (C18:0), oleic acid (C18:1), linoleic acid (C18:2) and α-linolenic acid (C18:3) (Fig. 6a). The analysis of fatty acid composition revealed that the expression of *At-Gh13LPAAT5* in the yeast led to an approximately 25–31% increase in palmitic acid (C16:0) and oleic acid (C18:1) when compared to the non-transgenic control strains (Fig. 6b), and no significant differences were detected between the transgenic yeasts with genes from the Upland and *G. barbadense* sources. As compared to the empty vector transformants, the total TAGs in the transgenic strains were also increased within the range of 16–29%, and transgenic yeasts with genes from both sources also showed similar results. The qRT-PCR analysis showed that the gene expression pattern was essentially similar to the concentration of TAGs among the three transgenic yeast strains tested (Fig. 6 c, d). These results indicate that expression of the foreign gene *At-Gh13LPAAT5* in the yeast can enhance oil content and selectively incorporate fatty acids into TAGs, and the gene alleles from both the Upland and *G. barbadense* sources has a similar effort.

## Discussion

### The *LPAAT* gene family in *Gossypium*

The results of this study revealed details of 40 *LPAAT* genes in the multigene family in four *Gossypium* species with sequenced genomes: diploids *G. raimondii* and *G. arboreum*, and their descendant tetraploids *G. hirsutum* and *G. barbadense*. The translated protein sequences were clustered into four groups (subfamilies), consistent with other previous reports [20]. Consistent with previous studies that whole genome duplication events occurred during the evolution of *Gossypium* [37], we observed that each *GrLPAAT* gene or *GaLPAAT* gene in each of the diploid species corresponded to two *GhLPAAT* genes in the tetraploid cotton belonging to one homeologous *LPAAT* group. The eleven putative plastidial LPAATs in cotton showed a high similarity to the previously characterized phospholipid/glycerol acyltransferase family proteins from coconut. Proteins in the A-class LPAAT (LPAAT2/3) group, the B-class LPAAT group, and the plastidial LPAAT group were shown to have LPAAT activity [20]. However, in *Arabidopsis*, members of the LPAAT4/5 subfamily did not show LPAAT activity in *vitro* [25].

In this study, *At-Gh1LPAATB*, *Dt-Gh1LPAATB*, *At-Gb1LPAATB*, *Dt-Gb1LPAATB*, *Ga4LPAATB*, and *Gr2LPAATB*, were predicted to be in the B-class LPAAT group in cotton. Previous reports suggested that the B-class proteins are seed-specific isoenzymes, such as *LdLPAATB* and *CnLPAATB* isolated from poached eggplant and coconut, respectively [19, 22]. However, recent studies have shown that B-class proteins are not restricted to plant seeds. *RcLPAATB* (formerly called *RcLPATB*) identified in castor, was shown to express at similar levels in other tissues/organs of the plant [20]. We obtained a similar result in that there were very low transcript levels of B-class *LPAATs* in ovules based on our RNA-seq analyses. This is possibly because the RNA-seq data were obtained at the fiber initiation stage, and not the seed oil accumulation stage. The qRT-PCR results showed that *Dt-Gh1LPAATB* did not show a typical B-class expression pattern, but was expressed throughout the plant.

### Expression of *LPAAT* genes and their natural variations in relation to seed oil content and fiber development

In higher plants, LPAATs catalyze the incorporation of acyl groups into the *sn*-2 position of LPAs to yield PAs, the key intermediates in the synthesis of membrane phospholipids or TAG storage lipids [11]. Each cotton fiber is a single cell that can be used as a biological model to study cell expansion. During the rapid polarized expansion of the fiber cell, abundant membrane phospholipids are required to synthesize the cytomembrane so that the fiber cell can elongate from 10 to 15 mm at 5–10 DPA to 25–30 mm at 25 DPA. Cottonseed oil accumulates later in ovules after 15–20 DPA. Therefore, in developing cottonseeds, LPAATs maybe play a dual role in that they produce raw materials for the biosynthesis of the membrane as the cell elongates and also catalyze the conversion of LPAs to TAGs. To test this hypothesis, we analyzed *LPAATs* in Xuzhou 142 and Xuzhou 142 *fl*, which have a similar genetic background based on the origin of the mutant. Xuzhou 142 *fl,* the natural fuzzless-lintless mutant from the fibered wild type Xuzhou 142 with both lint and fuzz, not only has few or no fibers, but also has higher seed oil content than that of Xuzhou 142 [42].

The qRT-PCR results revealed different expression patterns of *LPAAT* genes, even those in the same subfamily. These findings suggested that *LPAAT* genes have diverse developmental expressions. Based on their expression patterns, the *LPAAT*s were grouped into two categories. The first category (*At-Gh6LPAAT1*, *At-Gh1LPAAT2*, *At-Gh1LPAAT3*, *At-Gh13LPAAT5* and *Dt-Gh1LPAATB*) showed high expression levels at two significant phases, i.e., during the rapid phase of fiber elongation (10 DPA) in Xuzhou 142 and at the beginning of fatty acid accumulation in ovules of Xuzhou 142 *fl* (20 DPA). At 10 DPA, fiber cells require membrane phospholipids to form new membranes. It is possible that these genes were preferentially expressed during membrane biosynthesis in cotton. Interestingly, these genes showed a sharp increase in transcript levels in the 20 DPA Xuzhou 142 *fl* ovules, but slower increases in transcript levels in Xuzhou 142 at 20 DPA ovules and during other stages of Xuzhou 142 *fl* ovule development. This may be because the fuzzless/lintless mutant Xuzhou 142 *fl* seed has few or no fibers, and therefore, does not require large amounts of membrane phospholipids for new cytomembranes of fiber development. In that case, the LPAATs would synthesize TAGs for seed oil production, rather than membrane phospholipids for developing fibers. The second group of genes (*At-Gh5LPAAT1*, *At-Gh6LPAAT1*, *Dt-Gh9LPAAT1* and *Dt-Gh6LPAAT4*) was expressed preferentially in fiber tissues, especially *Dt-Gh6LPAAT4*. Their expression levels were very low in ovules of both Xuzhou 142 and Xuzhou 142 *fl*. This result suggested that these three *LPAATs* in this group may be specific to membrane biosynthesis, but more studies are needed. However, it should also be pointed out that, the expression magnitudes at the RNA level might not necessarily reflect the expression of these genes at the enzymatic level. In this study, the expression patterns of the *LPAAT* genes between the two genotypes were overall similar, and no SNPs of *LPAATs* identified and developed from a comparison between the two lines were located within the two gene regions for the fiberless and fuzzless traits [41]. We therefore conclude that none of the *LPAAT* genes was directly involved with the fiberless and fuzzless mutation and its higher oil content in Xuzhou 142 *fl*. Therefore, a segregating population between the two genotypes is unnecessary to address the role of *LPAATs* in the molecular mechanism of the fiberless and fuzzless trait in this mutant.

To further test if *LPAAT* genes are related to cotton fiber elongation, a pair of BILs with different fiber length and similar seed oil content was used. Our results indicated that the expression levels of *LPAAT* genes were similar between the two lines, suggesting that the genetic differences in fiber elongation between the two NILs are not associated with the expression of *LPAAT* genes. Furthermore, a physical mapping of *LPAAT* genes with QTL for cottonseed oil content showed that 10 of the 13 *LPAAT* genes were located within QTL regions (i.e., within 25 cM) for seed oil or protein content. However, to further test if *LPAAT* genes are genetically related to cottonseed oil production and fiber quality traits, a backcross inbred line population (from which the two BILs were selected) of 146 lines differing in fiber quality traits and seed oil content and fiber quality were used. The results indicated that, of all the potential SNPs identified in the *LPAAT* genes, only one marker from one gene *At-Gh13LPAAT5* showed a significant correlation with cottonseed oil content and fiber uniformity in only one of the three field tests, suggesting that sequence differences in most if not all of the *LPAAT* genes are not involved in the natural genetic variation for seed oil production and fiber quality traits in cotton.

Furthermore, we designed an experiment to see if the two *LPAAT* variants of the same gene conferred by a SNP (which differed in a synonymous base change) had a similar effect on oil content and composition in a yeast system. Both variants were supposed to produce the protein with the same sequence and therefore a similar biochemical activity. As expected, the transgenic yeast experiment showed that the total TAG and palmitic acid and oleic acid increased compared to the nontransgenic yeast control, similar to these obtained in other transgenic plant studies [28]. The results further showed that both sources of the *At-Gh13LPAAT5* gene had a similar effect, proving the lack of association between the natural variation of the *LPAAT* gene and fatty acid content and composition.

## Conclusions

A total of 40 *LPAAT* genes were identified and grouped into four distinct subfamilies in four *Gossypium* species, i.e., i.e*.*, tetraploid *G. hirsutum*- AD_1_ and *G. barbadense*- AD_2_ and its ancestral diploids *G. raimondii*- D_5_ and *G. arboreum*- A_2_. The detailed analysis of the sequence variation, QTL co-localisation and content of TAG in transgenic yeasts showed that natural sequence variations in the *LPAAT* genes are highly limited which are unlikely associated with the natural variations in seed oil and protein contents and fiber quality traits in cotton. However, the cotton *LPAATs* can increase oil composition and content as demonstrated in the transgenic yeast experiment. The results provide an important lead for further studies to elucidate the involvement of *LPAAT* genes in the natural variation of cottonseed oil content and a possible strategy in genetic engineering for the improvement of seed oil content and composition in cotton.

## Methods

### Materials

Xuzhou 142 (wild type), an Upland cotton cultivar and its fuzzless/lintless natural mutant (Xuzhou 142 *fl*) differing in fiber initiation, and two backcross inbred lines (NMGA-062 with fiber length of 32.58 mm and NMGA-105 with fiber length of 27.06 mm) differing in fiber length, were used for RNA-seq and tissue/organ quantitative real-time RT-PCR analysis. Xuzhou142 *fl* and its wild type also had significantly different oil contents and fatty acid compositions [42]. Xuzhou 142 and Xuzhou 142 *fl* were grown at the Institute of Cotton Research (ICR), Chinese Academy of Agricultural Sciences (CAAS), Anyang, Henan province, China. On selected days post anthesis (DPA), ovules were excised from developing bolls, and fibers were scraped from the epidermis of the ovules. Petals were sampled on the day of flowering, and roots, stems and leaves were also collected from seedlings at 2 weeks after germination. All samples were quick-frozen in liquid nitrogen and stored at −80 °C until use. For each time-point or tissue, three biological replications were collected.

Since the other cultivated tetraploid cotton *G. barbadense* contains higher seed oil content and lower protein content than *G. hirsutum*, introgression lines between the two species were also used in this study. SG 747 (*G. hirsutum*) was first crossed with Giza 75 (*G. barbadense*), and the resulting F_1_ was backcrossed with SG 747 twice followed by three generations of self-pollination to produce advanced backcross inbred lines (BILs). The two parents and 146 BILs including NMGA-062 and NMGA-105 were grown in two replications using a randomized complete block design in Anyang, Henan in 2006, 2008, and 2009. Crop management practices and boll sampling followed local recommendations [44]. Mature seed after ginning boll samples was determined for oil and protein contents using the near infrared reflectance spectroscopy [44, 45], which were also used in other cotton studies [46, 47]. Another non-destructive measuring techniques, i.e., nuclear magnetic resonance [48] was not used in the current study. Fiber quality traits were measured using High Volume Instrument (HVI) 1000 by ICR, CAAS, Anyang, Henan [49].

To study the gene function of the heterologous gene, *At-Gh13LPAAT5* from both Upland cotton and *G. barbadense* sources, the methylotrophic yeast, *Pichia pastoris* was used. The *P. pastoris* strain GS115 and plasmid pPIC3.5 K were kept in our laboratory.

### Prediction and cladistic analyses of *LPAAT* genes

The genome sequences of *G. arboreum*, *G. raimondii*, *G. hirsutum* and *G. barbadense* were downloaded from CottonGen database (https://www.cottongen.org/home). Putative *LPAATs* were identified in the PFAM protein family database using HMMER software version 3.0 [50], with the LPAAT domain (PF01553, corresponding to LPLAT_LCLAT1-like and LPLAT_AGPAT-like in NCBI CDD) as the search query [51], with an initial threshold value of *E* ≤ 10^−20^. For the cladistics analysis of LPAAT*-*like proteins, the representative LPAAT protein sequences from other species including the model plant *Arabidopsis*, and *T. cacao*, which shared an ancestor with cotton at least 60 million years ago [35], were downloaded from the NCBI website (http://www.ncbi.nlm.nih.gov/guide/). Amino acid sequences were aligned using Clustal X v. 2.0.11 (http://www.clustal.org/) under the default settings, and were further refined by a visual inspection. The alignment outputs were used to construct cladograms using the Neighbor-Joining (NJ) method, as implemented in MEGA v. 5 (http://www.megasoftware.net/). The bootstrap consensus tree was inferred from 1000 replicates.

### In-silico mapping and analysis of *LPAAT* genes

Mapping of *LPAAT* genes was performed using MapChart (http://www.earthatlas.mapchart.com/) [52]. Quantitative trait loci (QTL) in this paper were downloaded from CottonQTLdb (http://www.cottonqtldb.org) [43, 53, 54]. The genetic structures of *LPAAT* genes were generated using the GSDS (Gene Structure Display Server) algorithm (http://gsds.cbi.pku.edu.cn/). All of the putative protein sequences were analyzed using the domain analysis program CDD (http://www.ncbi.nlm.nih.gov/Structure/cdd/wrpsb.cgi) with the default cut-off parameters. The length, molecular weight, and isoelectric point of each LPAAT protein were calculated using ExPasy (http://web.expasy.org/compute_pi/). The subcellular localization of each protein was analyzed using PSORT Prediction (http://psort.hgc.jp/form.html).

### Analysis of *LPAAT* genes in two RNA-seq datasets

To study the expression of *LPAAT* genes in ovules and fibers during fiber initiation and elongation stages, the transcriptional profiles of *LPAAT* genes were analyzed using information from two transcriptome sequencing databases. One database contained transcriptomic information for 0, 3 and 10 DPA ovules for the following two backcross inbred lines (BILs) with significantly different fiber lengths but similar oil and protein contents in seed: NMGA-062 and NMGA-105. The other database contained transcriptomic information for −3 and 0 DPA ovules of the cotton cultivar Xuzhou 142 and its natural fiberless mutant Xuzhou 142 *fl*. These databases are generated in our own laboratory and can be accessed through NCBI under accession numbers SRP038911, SRP039385, and SRP056184. Transcriptome analyses were conducted with MeV software [55].

### RNA isolation and real-time PCR analysis

Based on the *LPAAT* coding sequences, gene-specific primers for qRT-PCR were designed with Oligo 7 software (Additional file 3: Table S1). Because we attempted to study the differential expression of *LPAATs* in cotton fiber and seed oil, total RNA was isolated from ovules at the fiber initiation stage (−3, −1 and 0 DPA), ovules and fibers at the early fiber elongation stage (3–10DPA), and ovules and fibers at the seed oil accumulation stage (20–25DPA). Other cotton tissues including roots, stems, leaves, and petals were also sampled and analyzed for a comparison. Each sample possessed 3 biological replicates. The RNA Prep Pure Plant kit (Tiangen, Beijing, China) was used to extract RNA. Then, 0.5 μg purified total RNA was reverse-transcribed into cDNA using the Super Script First-Strand Synthesis System for RT-PCR (PrimeScript, Takara, Dalian, China), following the manufacturer's instructions. Because of high homologies among *LPAAT* gene sequences within the same subfamily, 8 typical *LPAAT* genes from four gene subfamilies were chosen. The transcript levels of the 8 *LPAAT* genes were normalized to the mean value of *Histone*3 (AF024716) used as an internal control. All qRT-PCR reactions were run on an iCycler iQ5 Fast Real-Time PCR System (Bio-Rad, Hercules, CA, USA) according to the manufacturer’s instructions. Each 20 μL reaction mixture contained 10 μL SYBR Premix Ex *Taq* II (2×), 0.4 μL forward and reverse primers (10 μM), 2 μL diluted cDNA, and ddH_2_O up to 20 μL. The thermal profile used for all PCRs was as follows: 10 min at 95 °C for DNA polymerase activation, 40 cycles of 15 s at 95 °C, 30 s at 58 °C, and 20 s at 72 °C, and then a 5 min elongation step at 72 °C. The default settings were selected for the melting curve analysis. The gene transcript levels were calculated using the 2^-△△CT^ method. Three biological replicates, each with three technical replicates, were evaluated.

### Identification of single nucleotide polymorphisms (SNPs) for *LPAAT* genes and statistical analysis with cottonseed oil and protein content and fiber quality traits

The two RNA-seq datasets were also used to identify SNPs for *LPAAT* genes. Assembled contigs (unigenes) were scanned for SNPs using SNP detection software SOAPsnp [56]. Putative SNPs were identified to design primers (Additional file 3: Table S2) using Oligo7 for developing single strand conformation polymorphic (SSCP) markers for the BIL population per the method of Lu et al. [57]. The SSCP markers were coded as “1” (i.e., *G. hirsutum* or *G. barbadense* allele) for presence and “0” for absence of a SSCP marker and used to perform a simple correlation analysis with the seed oil and protein contents in the BIL population using SPSS software (IBM, New York, USA).

### Construction of the *At-Gh13LPAAT5* yeast expression vector and yeast transformation

As a SSCP marker developed from the gene *At-Gh13LPAAT5* was correlated to cottonseed oil content, the gene was used in this study. First-strand cDNA synthesis was carried out using ReverTra Ace qPCR RT Master Mix (Toyobo, Japan) from Upland TM-1, Xuzhou 142, Xuzhou 142 *fl* and *G. barbadense* 3–79 used as a template for PCR to amplify *At-Gh13LPAAT5* using the primer pair At-Gh13LPAAT5F/At-Gh13LPAAT5R (5'-ATTATTCGAAGGATCCATGGAAGTTCCAAGTGCGAAA-3'/ 5'-CCGCCCTAGGGAATTCTTAAGCTCCCGACATGAACC-3'), and the PCR products were sequenced in Genewiz for validation of the sequences. Because the sequences from Upland Xuzhou 142 *fl* (which was likely derived from a natural hybrid between Upland Xuzhou 142 and an unknown *G. barbadense* based on Ma et al. 2016) [41] and *G. barbadense* 3–79 were identical which were different from the sequence from Upland TM-1 and Xuzhou 142 (Additional file 8: Figure S1), the cDNA sequences encoding the At-Gh13LPAAT5 from Xuzhou 142 *fl* (to represent the *G. barbadense* gene allele) and Xuzhou 142 were separately cloned into the *EcoR*I site of the expression vector pPIC3.5 K [58, 59]. 50 μL competent cells of *Pichia pastoris* GS115 was transformed with 10 μg of *Sac*I-linearized pPIC3.5 K that was recombined with *At-Gh13LPAAT5* by LiCl according to the Invitrogen manual.

### Gas chromatography/mass spectrometry (GC/MS) profiling and statistical analysis

A GC/MS analysis was performed using a gas chromatograph (7890A, Agilent Technologies, USA) equipped with a flame ionization detector (FID) and an HP-FFAP capillary column (30 m × 250 μm × 0.25 μm) by OCRI-CAAS. High purity nitrogen was used as carrier gas. Inlet pressure is 25 psi,and sample volumes of 1 μL were injected with a split ratio of 30:1 using a hot-needle technique. The GC column temperature was programmed from 150 (initial equilibrium time, 1 min) to 230 °C via a ramp of 5 °C/min and maintained at 230 °C for 8 min. The injection temperature was 250 °C, and the test temperature was set to 280 °C [60]. Transgenic yeasts were induced for 72 h and internal standard gas chromatography was performed to determine the fatty acid components and the content of triacylglycerols (TAGs). The t-test was performed using the Microsoft excel.

## Additional files

Additional file 1: Figure S2. An alignment of LPAAT2/3 with LPAAT4/5 by ClustalX2 program. The boxes indicate variation. (TIF 2178 kb)
Additional file 2: Figure S3. An alignment of LPAAT1 with B-class LPAAT by ClustalX2 program. (TIF 4401 kb)
Additional file 3: Table S1. Sequences of primers used for studies of *GhLPAAT* expression. **Table S2.** Sequences of primers of *GhLPAATs* to identify single nucleotide polymorphic markers (SNPs) based on single strand conformation polymporphism (SSCP). **Table S3.** Domain analysis and annotation. **Table S4.** Oil and protein quantitative trait loci (QTL). **Table S5.** Identification of putative sequence variation (SNPs) by SOAPsnp in NMGA-062 vs. NMGA-105 database. L indicates SNP only exists in long fiber length line NMGA-062 (fiber length, 32.58 mm) when compared with the sequenced genome sequence of TM-1. S indicates SNP only exists in short fiber length line NMGA-105 (fiber length, 27.06 mm) when compared with the sequenced genome sequence of TM-1. L/S indicates SNP exists in the two lines. **Table S6.** Identification of putative SNPs by SOAPsnp in Xuzhou 142 vs. Xuzhou 142 *fl* database. WT indicates SNP only exists in Xuzhou 142 when compared with the sequenced genome sequence of TM-1. *fl* indicates SNP only exists in Xuzhou 142 *fl* when compared with the sequenced genome sequence of TM-1. WT/*fl* indicates SNP exists in the two lines. The polymorphic SSCP marker is shown in red color. (XLSX 26 kb)
Additional file 4: Figure S4. Chromosomal distribution of *LPAAT* genes. Dotted lines link homoelogous *LPAATs* between A_2_ and D_5_ chromosomes. The scale to the left of chromosomes denotes the size of chromosomes in million bases (Mb). (TIF 6248 kb)
Additional file 5: Figure S5. Expression patterns of *Dt-Gh1LPAATB* genes in other organs. *Histone*3 was used as an internal control. (TIF 46 kb)
Additional file 6: Figure S6. Sequence variations in the predicted *LPAAT* genes between the sequenced *G. hirsutum* (TM-1) and *G. barbadense* (3–79 and Xinhai 21). The boxes indicate the variations of the A- and D- subgenomes and the ellipses indicate the SNPs between the two cultivated species; Gb1 and Gb2 indicate 3–79 and Xinhai21 respectively. (PDF 22277 kb)
Additional file 7: Figure S7. A polymorphic SSCP marker developed from a *LPAAT* gene in the backcross inbred population of SG 747 x Giza 75. The arrow indicates the polymorphic marker. (TIF 9674 kb)
Additional file 8: Figure S1. An alignment of *At-Gh13LPAAT5* in four *Gossypium* species. The box indicates the variation of *At-Gh13LPAAT5* at 342th nucleotide. (TIF 2008 kb)

## Abbreviations

DGAT: Diacylglycerol acyltransferase
DPA: Days post-anthesis
GPAT: Glycerol-3-phosphate O-acyltransferase
LPA: Lysophosphatidate
LPAAT: Lysophosphatidic acid acyltransferase
LPC: Lysophosphatidylcholine
MUFAs: Monounsaturated fatty acids
PAs: Phosphatidic acids
PC: Phosphatidylcholine
PDAT: Phospholipid:diacylglycerol acyltransferase
PDCT: Phosphatidylcholine:diacylglycerol cholinephosphotransferase
SFAs: Saturated fatty acids
SNPs: Single nucleotide polymorphisms
SSCP: Single strand conformation polymorphic
TAGs: Triacylglycerols
Xuzhou 142 *fl*: fuzzless/lintless natural mutant of Xuzhou 142
