# Supplementary figures and images for: A genome-wide analysis of the lysophosphatidate acyltransferase (LPAAT) gene family in cotton: organization, expression, sequence variation, and association with seed oil content and fiber quality
Source: BMC Genomics. 2017 Mar 1;18:218. doi: 10.1186/s12864-017-3594-9 (PMC5333453; doi:10.1186/s12864-017-3594-9)

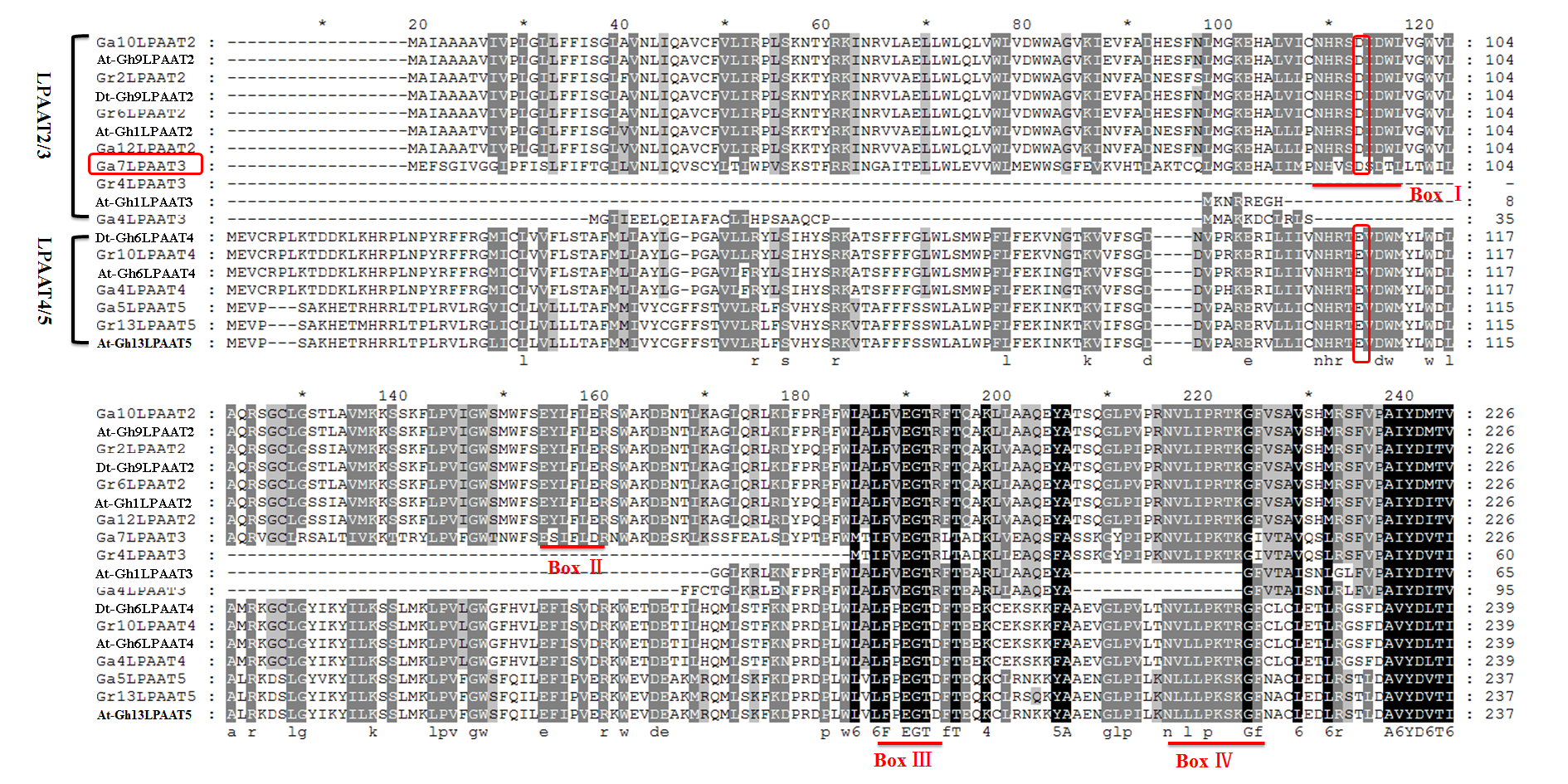

Supplement: Additional file 1: Figure S2. — An alignment of LPAAT2/3 with LPAAT4/5 by ClustalX2 program. The boxes indicate variation. (TIF 2178 kb) [file 12864_2017_3594_MOESM1_ESM.tif]

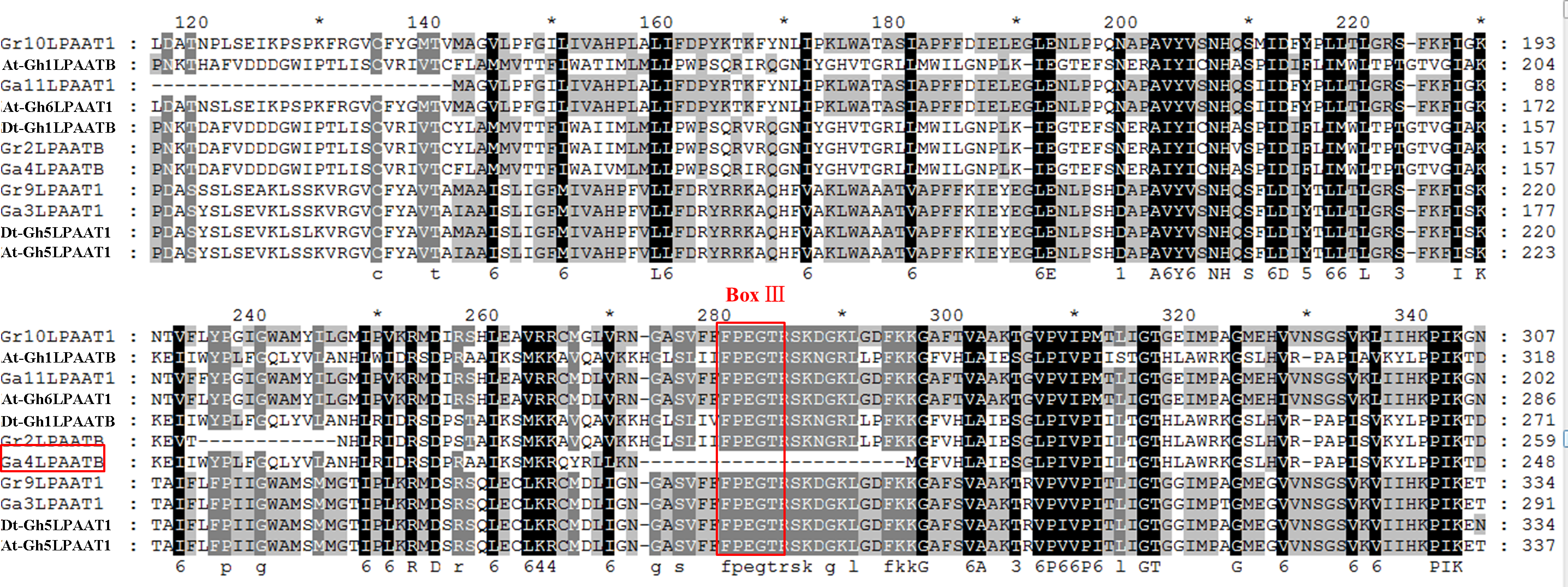

Supplement: Additional file 2: Figure S3. — An alignment of LPAAT1 with B-class LPAAT by ClustalX2 program. (TIF 4401 kb) [file 12864_2017_3594_MOESM2_ESM.tif]

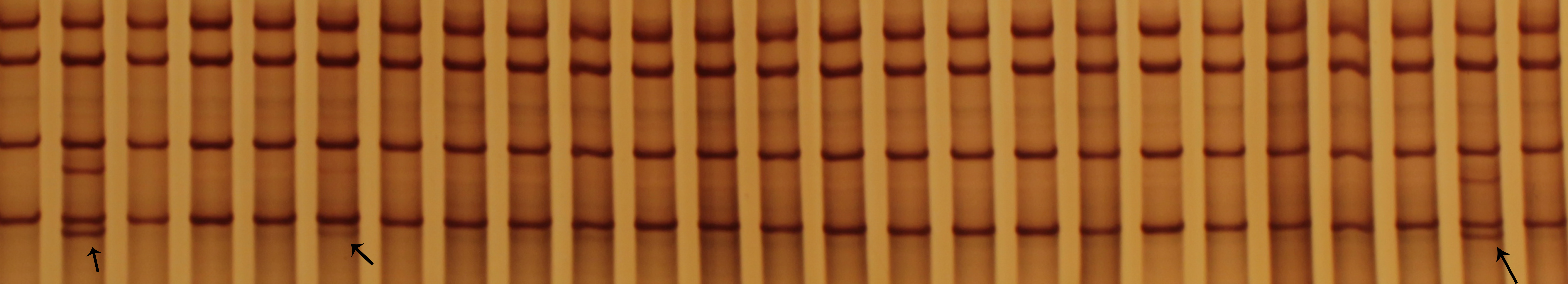

Supplement: Additional file 7: Figure S7. — A polymorphic SSCP marker developed from a LPAAT gene in the backcross inbred population of SG 747 x Giza 75. The arrow indicates the polymorphic marker. (TIF 9674 kb) [file 12864_2017_3594_MOESM7_ESM.tif]
