# Supplementary material for: A genome-wide analysis of the lysophosphatidate acyltransferase (LPAAT) gene family in cotton: organization, expression, sequence variation, and association with seed oil content and fiber quality
Source: BMC Genomics. 2017 Mar 1;18:218. doi: 10.1186/s12864-017-3594-9 (PMC5333453; doi:10.1186/s12864-017-3594-9)
Supplement: Additional file 6: Figure S6. — Sequence variations in the predicted LPAAT genes between the sequenced G. hirsutum (TM-1) and G. barbadense (3–79 and Xinhai 21). The boxes indicate the variations of the A- and D- subgenomes and the ellipses indicate the SNPs between the two cultivated species; Gb1 and Gb2 indicate 3–79 and Xinhai21 respectively. (PDF 22277 kb) [file 12864_2017_3594_MOESM6_ESM.pdf]

LPAAT1

```
                        *        20         *        40         *        60         *        80         *       100         *
A-Gh-LPAAT1  : ---------------------------------ATGCAACTCTCTACACTTTCTCTTTTCCCTTTCTGTAACTTCCTCACTCTCTCTATACACTTTCCCATCTACCTAATCC :   79
A-Gb2-LPAAT1 : ATGGAAGTTCCAAGTGCGAAACATGAAACAAGGCATCGTAGGTTGACCCCGCTTAGGGTGTTAAGGGGTCTGATATGTTTGCTAGTGTTGCTTTTAACAGCTTTTATGATGA :  112
A-Gb1-LPAAT1 : ---------------------------------------------------------------------------------------------------ATGCAACTCTCTA :   13
D-Gh-LPAAT1  : ---------------------------------------------------------------------------------------------------------------- :    -
D-Gb1-LPAAT1 : ---------------------------------ATGCAACTCTCTACACTTTCTCTTTTCCCTTTCTGTAACTTCCTCACTCTCT--ATACACTTTTCCATCTACCTAATCC :   77
D-Gb2-LPAAT1 : ---------------------------------------------------------------------------------------------------------------- :    -


                       120         *       140         *       160         *       180         *       200         *       220
A-Gh-LPAAT1  : TATCTTCTTCTTTATGCAAAACTGTTCCCAAATTTCTGCATATTAAGGCGAGCCGTGAGCGTGAA-TCTCGGTGTTATTTTACTCTAAATCAGGGATTCAATAGATCAAACA :  190
A-Gb2-LPAAT1 : TAGTGTATTGTGGCTTTTTTAGTACTGTTGTATTGAGGCTTTTCAGCGTACATTACAGCCGAAAAGTAACAGCTTTCTTCTTTAGTTCTTGGTTAGCATTATGGCCTTTTCT :  224
A-Gb1-LPAAT1 : CACTTTCTCTTTTCCCTTTCTCTGTTCCCAAATTTCTGCATATTAAGGCGAGCCGTGAGGGTGAA-TCTCGGTGTTATTTTACTCTAAATCAGGGATTCAATAGATCAAACA :  124
D-Gh-LPAAT1  : -------------------------------------------------------------------------------------------------ATGCTGCAACGCGTG :   15
D-Gb1-LPAAT1 : TAT-TTCTTCTTTATGCAAAACTGTTCCTAAATTTCTGCATATTAAGGCGAGCCGTGAGGGTGAA-TCTCGGTGTTATTTTACTCTAAATCAGGGATTCAATCGATCAAACA :  187
D-Gb2-LPAAT1 : ---------------------------------------------------------------------------------------------------------------- :    -
                                                                                                                    t

                     *       240         *       260         *       280         *       300         *       320         *
A-Gh-LPAAT1  : AATTGTCCAGATACATAGTTACAAGATCTCAACTTGGTGGGACTGGGATCCTTGATGCTACCAATTCTTTATCAGAAATTAAGCCGAGTCCGAAATTTAGAGGAGTATGCTT :  302
A-Gb2-LPAAT1 : ATTTGAGAAAATAAACAAAACCAAGGTCATTTTTTCTGGAGATGATGTTCCTGCAAGGGAACGAGTTTACTTATTTGCAACCACAGAACTGAGGTTGACTGGA-TGTACCT :  335
A-Gb1-LPAAT1 : AATTGTCCAGATACATAGTTACAAGATCTCAACTTGGTGGGACTGGGATCCTTGATGCTACCAATTCTTTATCAGAAATTAAGCCGAGTCCGAAATTTAGAGGAGTATGCTT :  236
D-Gh-LPAAT1  : GATGCACTA--TATGTTGTAGCGAAATCTGAACTTGCTGGGGCTGGAACACCTGATGCTTCCTATTCTTTATCAGAAGTTAAACTATCTTTGAAAGTTAGAGGAGTATGCTT :  125
D-Gb1-LPAAT1 : AATTGTCCAGATACATCGTTATAAGATCTCAACTTGCTGGAACTGGGATCCTTGATGCTACCAATTCTTTATCAGAAATTAAGCCGAGCCCGAAATTTAGAGGAGTATGCTT :  299
D-Gb2-LPAAT1 : ---------------------------------------------------------------------------------------------------------------- :    -
                 t     a  ta          a  tc     tt   g           c   a g    c a t  tt t a      a          ga   t a  gga t t c t

                 340         *       360         *       380         *       400         *       420         *       440
A-Gh-LPAAT1  : CTATGGTATGACAGTTATGGCAGGCGTTTTACCGTTTGGGATACTGATAGTGGCTCATCCCTTGGCGCTCATCTTTGATCCCTACAAGACAAAATTTTACAATCTTATTCCA :  414
A-Gb2-LPAAT1 : GTGGGATCTTGCATTGCGGAAAGATTCCCTGGGATACATAAAGTACATCCTTAAGAGCAGCTTAATGAAATTACCTGTATTTGGTTGGTCA----TTCCAAATTTTGGAGTT :  443
A-Gb1-LPAAT1 : CTATGGTATGACAGTTATGGCAGGCGTTTTACCGTTTGGGATACTGATAGTGGCTCATCCCTTGGCGCTCATCTTTGATCCCTACAAGACAAAATTTTACAATCTTATTCCA :  348
D-Gh-LPAAT1  : TTATGCTGTCACGGCAATGGCAGCTATTTCGCTTATTGGGTTTATGATAGTGGCACATCCCTTTGTGCTCTTATTTGATCGCTACAGAAGAAAAGCTCAGCATTTTGTTGCC :  237
D-Gb1-LPAAT1 : CTATGGTATGACAGTTATGGCAGGCGTTTTACCGTTTGGGATCCTGATAGTGGCTCATCCCTTGGCGCTCATCTTTGATCCCTACAAGACAAAATTTTACAATCTTATTCCA :  411
D-Gb2-LPAAT1 : ---------------------------------------------------------------------------------------------------------------- :    -
                t  g t t  c      g  ag                       at  t         ctt   g    t   tg             a     t    at tt

                 *       460         *       480         *       500         *       520         *       540         *       560
A-Gh-LPAAT1  : AAACTATGGGCAACCGCAAGTATTGCTCCCTTCTTCGATATCGAGTTGGAAGGATTGGAGAATCTGCCCCCGCAGAATGCTCCTGCGGTGTATGTTTC-----GAACCACCA :  521
A-Gb2-LPAAT1 : CATCCCTGTGGAAAGGAAGTGGGAGGTTGATGAGGCAAAAATGCGCCAGATGCTTTCAAAATTCAAAGATCCTCGAGATCCCCTTTGGCTTGTTCTCTTTCCAGAAGGAACA :  555
A-Gb1-LPAAT1 : AAACTATGGGCAACCGCAAGTATTGCTCCCTTCTTCGATATCGAGTTGGAAGGATTGGAGAATCTGCCCCCGCAGAATGCTCCTGCGGTGTATGTTTC-----GAACCACCA :  455
D-Gh-LPAAT1  : AAACTTTGGGCTGCAGCAACTGTTGCTCCGTTCTTTAAAATCGAGTATGAAGGATTGGAGAATTTGCCTTCACATGATGCTCCTGCCGTATATGTTTC-----CAACCACCA :  344
D-Gb1-LPAAT1 : AAACTATGGGCAACCGCAAGTATTGCTCCCTTCTTCGATATCGAGTTGGAAGGATTGGAGAATCTGCCCCCGCAGAATGCTCCTGCGGTGTATGTTTC-----GAACCACCA :  518
D-Gb2-LPAAT1 : ---------------------------------------------------------------------------------------------------------------- :    -
                a c  tg g     g a      g t   t      a a  g g   ga g  tt  a a t       c        c cct   g  t t  t        aa  a ca

                         *       580         *       600         *       620         *       640         *       660         *
A-Gh-LPAAT1  : GAGTATCATAGACTTCTATCCGCTGTTAACTCTGGGAA-GAAGCTTCAAGTTCATCGGCAAGAATACGGTATTTCTTTATCCTGGAATCGGGTGGGCC--ATGTATATTCTG :  630
A-Gb2-LPAAT1 : GATTTCACTGAGCAGAAGTGCTTAAGAAATAAAAAATATGCAGCTGAAAATGGCTTGCCTATCCTAAAGAATTTGCTGCTTCCAAAATCAAAGGGTTTTAATGCCTGCTTGG :  667
A-Gb1-LPAAT1 : GAGTATCATAGACTTCTATCCGCTGTTAACTCTGGGAA-GAAGCTTCAAGTTCATCGGCAAGAATACGGTATTTCTTTATCCTGGAATCGGGTGGGCC--ATGTATATTCTG :  564
D-Gh-LPAAT1  : GAGTTTTCTAGATATCTATACACTTCTAACTCTTGGAA-GAAGCTTCAAGTTCATCAGCAAGACTGCCATATTTCTTTTTCCCATTATTGGGTGGGCC--ATGTCTATGATG :  453
D-Gb1-LPAAT1 : GAGTATCATAGACTTCTATCCACTGTTAACTCTGGGAA-GAAGCTTCAAGTTCATCGGCAAGAATACGGTATTTCTTTATCCTGGAATCGGATGGGCT--ATGTATATTCTG :  627
D-Gb2-LPAAT1 : ---------------------------------------------------------------------------------------------------------------- :    -
               ga t    t         t c      aa        a g agct  aa t   t   c a   t     attt  t  t c    at     gg     atg  t     g

                     680         *       700         *       720         *       740         *       760         *       780
A-Gh-LPAAT1  : GGTATGATTCCGGTAAAGCGCATGGACATC-AGAAGCCATTTGGAGGCTGTTAGGAGATGTATGGATCTGGTAAGGAATGGTGCCTCCGTGTTTTTCTTTCCAGAAG---GA :  738
A-Gb2-LPAAT1 : AAGATTTGAGGAGCACTTTGGATG-CAGTTTACGATGTGACCATTGGATATAAGCATCGCTGTCCATC-ATTCTTGGATAACGTCTTTGGGGTTGACCCTTCTGAAGTTCAT :  777
A-Gb1-LPAAT1 : GGTATGATTCCGGTAAAGCGCATGGACATC-AGAAGCCATTTGGAGGCTGTTAGGAGATGTATGGATCTGGTAAGGAATGGTGCCTCCGTGTTTTTCTTTCCAGAAG---GC :  672
D-Gh-LPAAT1  : GGGACTATTCCATTAAAGCGCATGGACAGC-AGAAGCCAGTTGGAGTGTCTTAAGAGATGTATGGATCTCATCGGGAATGGTGCCTCTGTTTTTTTCTTTCCAGAAG---GC :  561
D-Gb1-LPAAT1 : GGTATGATTCCGGTAAAGCGCATGGACATC-AGAAGCCATTTGGAGGCTGTTAGGAGATGTATGGATCTGGTAAGGAATGGTGCCTCCGTGTTTTTCTTTCCAGAAG---GA :  735
D-Gb2-LPAAT1 : ---------------------ATGATCATTTACGATGTGACCATTGGATATAAGCATCGCTGTCCTTC-ATTCTTGGATAACGTCTTTGGGGTTGACCCTTCTGAAGTTCAT :   90
                  a          a    g ATG  cat  A A           Gg T T Ag A    T T  aTC   T   G AT   G CT  G g TT  C  T C GAAG

                       *       800         *       820         *       840         *       860         *       880         *
A-Gh-LPAAT1  : ACCCGGAGTAAGGACGGCAAGTTAGGTGATTTTAAG------AAAGGTGCATTCACTGTTGCAGCAAAAACCGGAGTGC--CTGTAATACCTATGACCTT--AATCGGCACC :  840
A-Gb2-LPAAT1 : ATTCACATCAGGCGTGTTACCCTGGATGACATCCCGTTGTCCGAAAGGGAAGTAACTTCTTGGTTAATGGATACATTCCAACGTAAGGATCAATTGCTTTCTAATTTCAATT :  889
A-Gb1-LPAAT1 : ACCCGGAGTAAGGACGGCAAGTTAGGTGATTTTAAG------AAAGGTGCATTCACTGTTGCAGCAAAAACCGGAGTGC--CTGTAATACCTATGACCTT--AATCGGCACC :  774
D-Gh-LPAAT1  : ACACGGAGTAAGGATGGGAAGTTAGGTGATTTCAAG------GTGAGATCAT--ACTAG----------------------------------------------------- :  612
D-Gb1-LPAAT1 : ACCCGGAGTAAGGACGGCAAGTTAGGTGATTTTAAG------AAAGGTGCATTCACTGTTGCAGCAAAAACCGGAGTGC--CTGTAATACCTATGACCTT--AATCGGCACC :  837
D-Gb2-LPAAT1 : ATTCACATCAGGCGTGTTACCCTGGATGACATCCCGTTGTCCGAAAGTGAAGTA---------------------------------------------------------- :  144
               A  C  A  A G   G  A   T G TGA  T   G       aa G g A t act

                 900         *       920         *       940         *       960         *       980         *      1000
A-Gh-LPAAT1  : GGCGAAATCATGCCTGCAGGAATGGAGCATATCGTCAACTCAGGCTCCGTTAAACTCATCATTCATAAACCCATCAAAGGAAACGATCCCGAAATTTTACGCCTACTAGCTA :  952
A-Gb2-LPAAT1 : CCGAAGGTTATTTCCCTCTGCATGGAGCAGAAT-TGAACCTCTCTACAGTCAAGTGCCTCATAAACGTCATGCTGGTATTCATTCACAAGCAGCATCAAGAAGTGCTTGCCG : 1000
A-Gb1-LPAAT1 : GGCGAAATCATGCCTGCAGGAATGGAGCATGTCGTCAACTCAGGCTCCGTTAAACTCATCATTCATAAACCCATCAAAGGAAACGATCCCGAAATTTTACGCCTACTATCTA :  886
D-Gh-LPAAT1  : ---------------------------------------------------------------------------------------------------------------- :    -
D-Gb1-LPAAT1 : GGCGAAATCATGCCTGCAGGAATGGAGCATGTCGTCAACTCAGGCTCCGTCAAACTCATCATTCATAAACCCATCAAAGGAAACGATCCCGAAATTTTACGCCAACTAGCTA :  949
D-Gb2-LPAAT1 : ------------------------------------------------------------------------------TTCATTCACAAGCAGCATCAAGAAGTGCTTGCTG :  178
                                                                                                a   a     a   t  a      ct  c

                 *      1020         *      1040         *      1060         *      1080         *
A-Gh-LPAAT1  : GAAACACCATTTTAGATACCTTAAGCATCGAGGCTCAGAAAAAAAAAACGGCTCTCATAAGCTCAAAACCATCTATCATTTAA : 1035
A-Gb2-LPAAT1 : GAAAAACTTCATTTGACATGACTTCCAGCATCATCCAAATGGGAAGG-TTCATGTCGGGAGCTTAA----------------- : 1065
A-Gb1-LPAAT1 : GAAACACCATTTTAGATACCTTAAGCATCGAGGCTCAGAAAAAAAAAACGGCTCTCATAAGCTCAAAACCATCTATCATTTAA :  969
D-Gh-LPAAT1  : ----------------------------------------------------------------------------------- :    -
D-Gb1-LPAAT1 : GAAACACCATTTTAGATACCTTAAGCATCGAGGCTCAGAAAAAAAAA-CGGCTCTCATAA----------------------- : 1008
D-Gb2-LPAAT1 : GAAAAACTTCATTTGACATGACTTCCAGCATCATCCAAATGGGGAGG-TTCATGTCGGGAGCTTAA----------------- :  243
               gaaa ac    tt ga a       ca c      ca a     a      t tc   a
```

LPAAT2

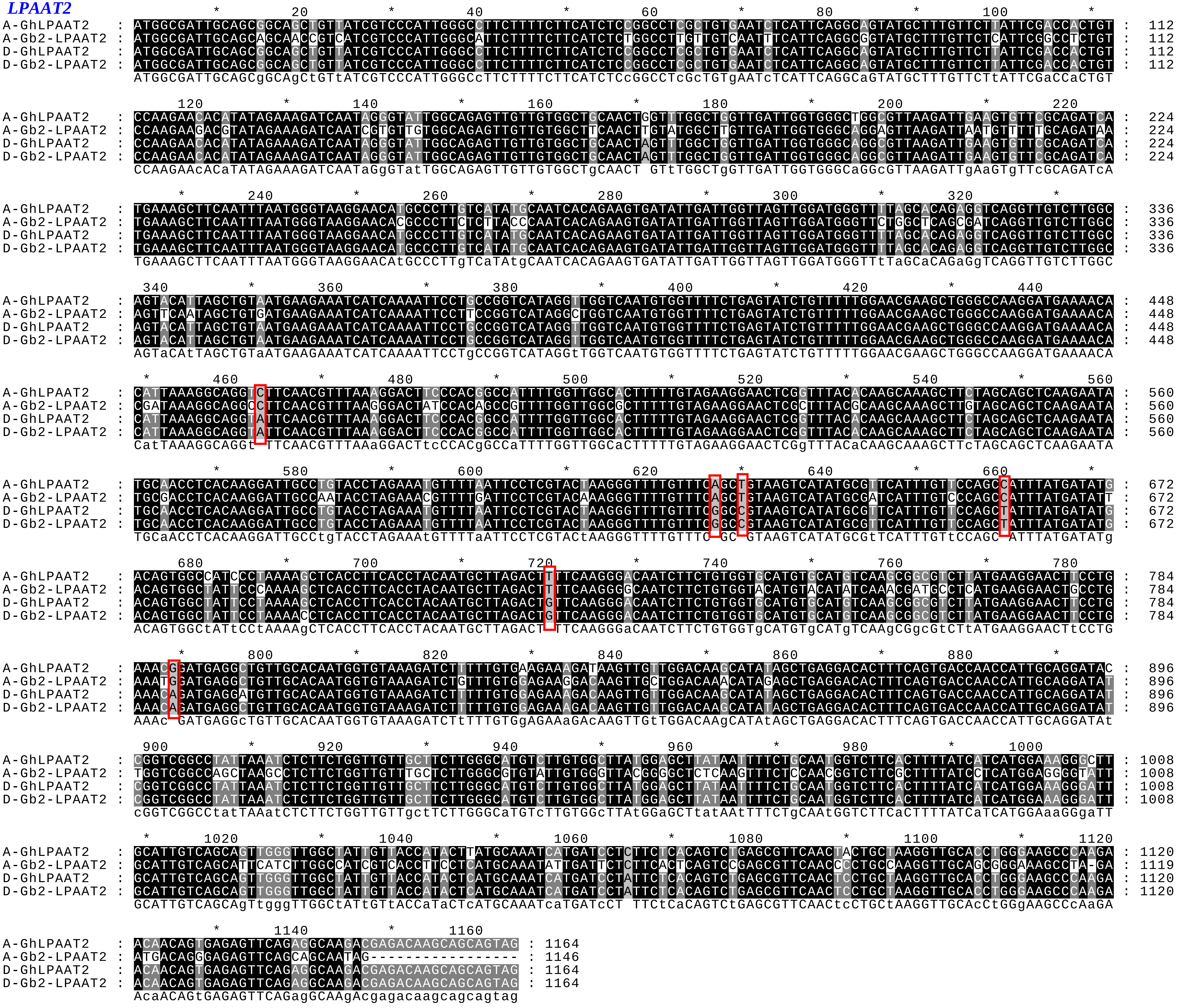

LPAAT4

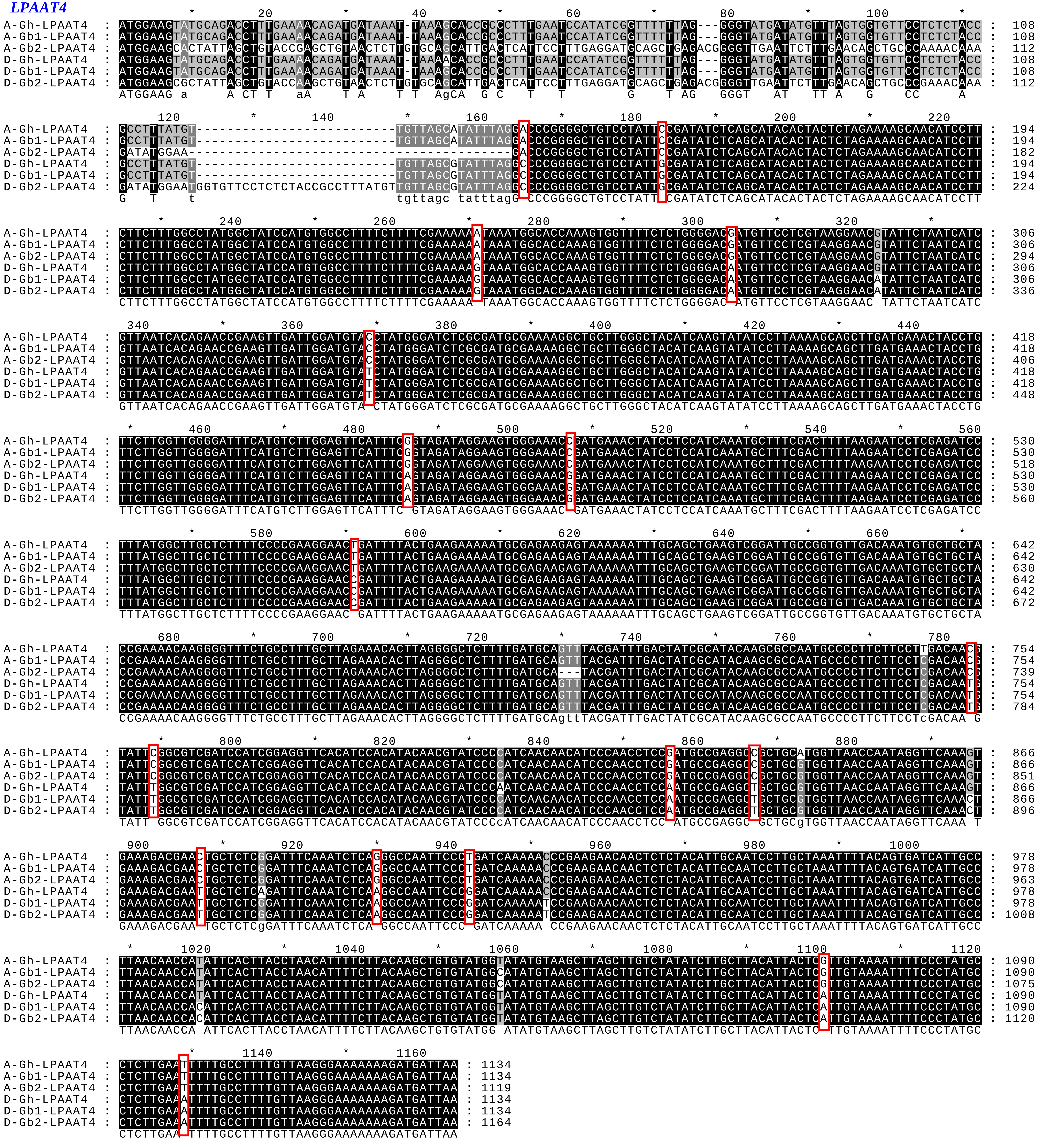
A-Gh-LPAAT4
A-Gb1-LPAAT4
A-Gb2-LPAAT4
D-Gh-LPAAT4
D-Gb1-LPAAT4
D-Gb2-LPAAT4

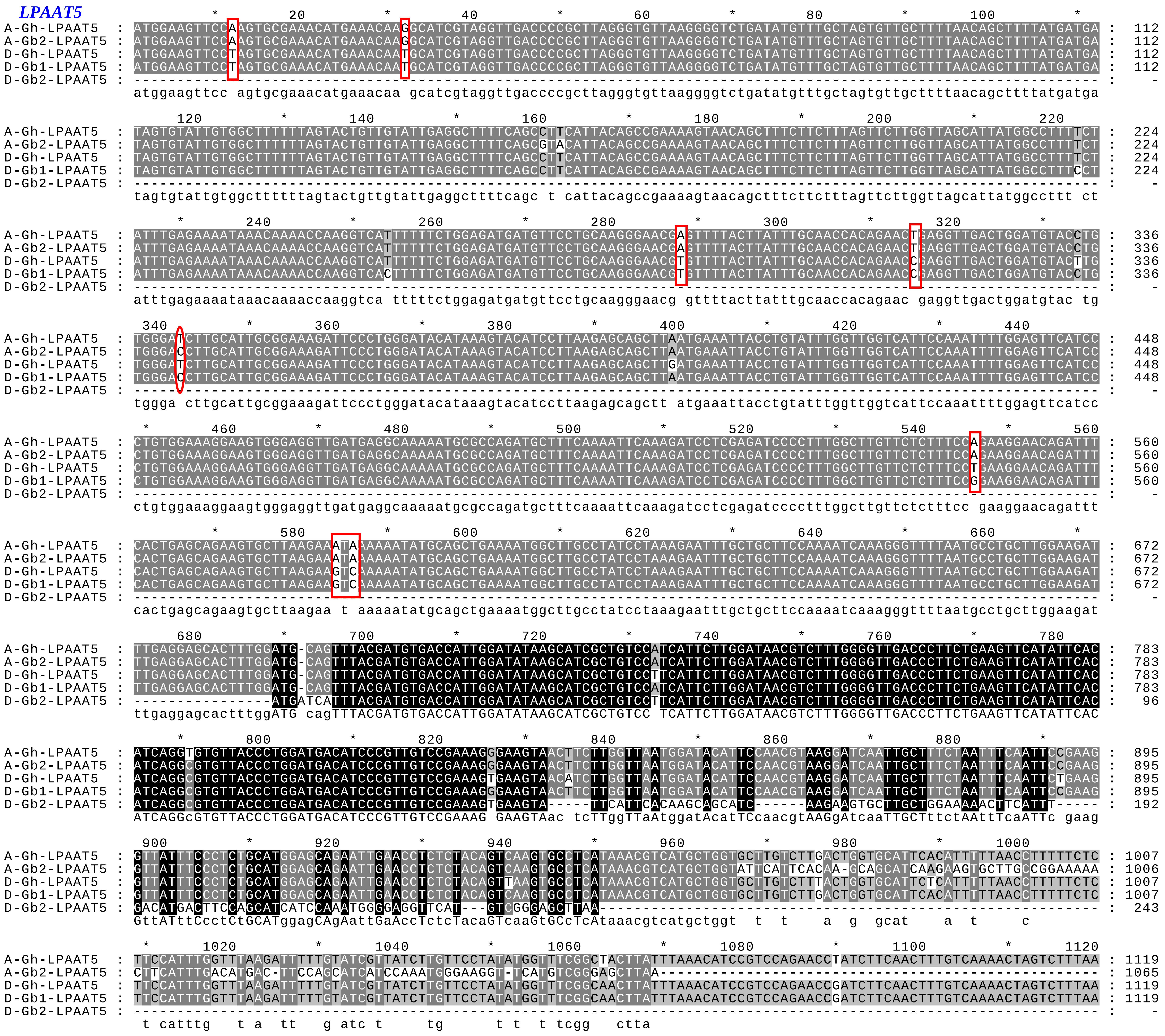
LPAAT5
A-Gh-LPAAT5
A-Gb2-LPAAT5
D-Gh-LPAAT5
D-Gb1-LPAAT5
D-Gb2-LPAAT5

LPAATB

```
                       *        20        *        40        *        60        *        80        *       100        *
A-Gh-LPAATB  : ATGGAGAACTCTGCAAGTGGTTCTTTTTTAAGGAACAGAAGATTAGAGAGCTTCCTCGAAACAAAATCTGGTCCAAATGTTAGAGAAACTCAAAAGATACCTAGCAAACAAGT : 113
A-Gb1-LPAATB : ATGGAGAACTCTGCAAGTGGTTCTTTTTTAAGGAACAGAAGATTAGAGAGCTTCCTCGAAACAAAATCTGGTCCAAATGTTAGAGAAACTCAAAAGATACCTAGCAAACAAGT : 113
A-Gb2-LPAATB : ATGGAGAACTCTGCAAGTGGTTCTTTTTTAAGGAACAGAAGATTAGAGAGCTTCCTCGAAACAAAATCTGGTCCAAATGTTAGAGAAACTCAAAAGATACCTAGCAAACAAGT : 113
D-Gh-LPAATB  : ATGGAGAACTCTGCAAGTGGTTCTTTTTTAAGGAACAGAAGATTAGAGAGCTTCCTCGAAACAAAATCTGGTCCAAATGTTAGAGAAACTCAGAAGATACCTAGCAAACAAGT : 113
D-Gb1-LPAATB : ATGGAGAACTCTGTAAGTGGTTCTTTTTTAAGGAACAGAAGATTAGAGAGCTTCCTCGAAACAAAATCTGGTCCAAATGTTAGAGAAACTCAGAAGATACCTAGCAAACAAGT : 113
D-Gb2-LPAATB : ATGGAGAACTCTGTAAGTGGTTCTTTTTTAAGGAACAGAAGATTAGAGAGCTTCCTCGAAACAAAATCTGGTCCAAATGTTAGAGAAACTCAGAAGATACCTAGCAAACAAGT : 113
               ATGGAGAACTCTG AAGTGGTTCTTTTTTAAGGAACAGAAGATTAGAGAGCTTCCTCGAAACAAAATCTGGTCCAAATGTTAGAGAAACTCA AAGATACCTAGCAAACAAGT

                    120        *       140        *       160        *       180        *       200        *       220
A-Gh-LPAATB  : AATAACAAGACAGAGGCCAAATAAAACTCATGCCTTTGTTGATGATGATGGATGGATTCCAACATTGATATCTTGTGTAAGGATTGTAACATGTTTCCTGGCAATGATGGTCA : 226
A-Gb1-LPAATB : AATAACAAGACAGAGGCCAAATAAAACTGATGCCTTTGTTGATGATGATGGATGGATTCCAACATTGATATCTTGTGTAAGGATTGTAACATGTTTCCTGGCAATGATGGTCA : 226
A-Gb2-LPAATB : AATAACAAGACAGAGGCCAAATAAAACTGATGCCTTTGTTGATGATGATGGATGGATTCCAACATTGATATCTTGTGTAAGGATTGTAACATGTTTCCTGGCAATGATGGTCA : 226
D-Gh-LPAATB  : AATAGCAAGACAGAGGCCAAATAAAACCGATGCCTTTGTTGATGATGATGGATGGATTCCAACATTGATATCTTGTGTAAGGATTGTAACATGTTACCTGGCAATGATGGTCA : 226
D-Gb1-LPAATB : AACAACAAGACAGAGGCCAAATAAAACCGATGCCTTTGTTGATGATGATGGATGGATTCCAACATTGATATCTTGTGTAAGGATTGTAACATGTTACCTGGCAATGATGGTCA : 226
D-Gb2-LPAATB : AATAACAAGACAGAGGCCAAATAAAACTGATGCCTTTGTTGATGATGATGGATGGATTCCAACATTGATATCTTGTGTAAGGATTGTAACATGTTTCCTGGCAATGATGGTCA : 226
               AAtAaCAAGACAGAGGCCAAATAAAAC gATGCCTTTGTTGATGATGATGGATGGATTCCAACATTGATATCTTGTGTAAGGATTGTAACATGTT CCTGGCAATGATGGTCA

                  *       240        *       260        *       280        *       300        *       320        *       34
A-Gh-LPAATB  : CAACATTCATCTGGGCAACTATCATGCTCATGCTCCTTCCTTGGCCTTCTCAGCGAATTAGACAAGGAAATATTTATGGCCATGTCACTGGTAGATTGCTGATGTGGATATTA : 339
A-Gb1-LPAATB : CAACATTCATCTGGGCAACTATCATGCTCATGCTCCTTCCTTGGCCTTCTCAGCGAATTAGACAAGGAAATATTTATGGCCATGTCACTGGTAGATTGCTGATGTGGATATTA : 339
A-Gb2-LPAATB : CAACATTCATCTGGGCAACTATCATGCTCATGCTCCTTCCTTGGCCTTCTCAGCGAATTAGACAAGGAAATATTTATGGCCATGTCACTGGTAGATTGCTGATGTGGATATTA : 339
D-Gh-LPAATB  : CAACATTCATCTGGGCAATTATCATGCTCATGCTCCTTCCTTGGCCTTCTCAGCGAGTTAGACAAGGAAATATTTATGGCCATGTCACTGGTAGATTGCTGATGTGGATATTA : 339
D-Gb1-LPAATB : CAACATTCATCTGGGCAATTATCATGCTCATGCTCCTTCCTTGGCCTTCTCAGCGAGTTAGACAAGGAAATATTTATGGCCATGTCACTGGTAGATTGCTGATGTGGATATTA : 339
D-Gb2-LPAATB : CAACATTCATCTGGGCAACTATCATGCTCATGCTCCTTCCTTGGCCTTCTCAGCGAATTAGACAAGGAAATATTTATGGCCATGTCACTGGTAGATTGCTGATGTGGATATTA : 339
               CAACATTCATCTGGGCAA TATCATGCTCATGCTCCTTCCTTGGCCTTCTCAGCGA TTAGACAAGGAAATATTTATGGCCATGTCACTGGTAGATTGCTGATGTGGATATTA

               0        *       360        *       380        *       400        *       420        *       440        *
A-Gh-LPAATB  : GGAAATCCATTGAAGATCGAAGGAACGGAGTTCTCGAATGAAAGAGCCATTTATATCTGCAATCATGCATCTCCCATTGACATTTTCCTCATTATGTGGTTGACTCCGACCGG : 452
A-Gb1-LPAATB : GGAAATCCATTGAAGATCGAAGGAACGGAGTTCTCGAATGAAAGAGCCATTTATATCTGCAATCATGCATCTCCCATTGACATTTTCCTCATTATGTGGTTGACTCCGACCGG : 452
A-Gb2-LPAATB : GGAAATCCATTGAAGATCGAAGGAACGGAGTTCTCGAATGAAAGAGCCATTTATATCTGCAATCATGCATCTCCCATTGACATTTTCCTCATTATGTGGTTGACTCCGACCGG : 452
D-Gh-LPAATB  : GGAAATCCATTGAAGATCGAAGGAACGGAGTTCTCGAATGAAAGAGCCATTTATATCTGCAATCATGCATCTCCCATTGACATTTTCCTCATTATGTGGTTGACTCCGACCGG : 452
D-Gb1-LPAATB : GGAAATCCATTGAAGATCGAAGGAACGGAGTTCTCGAATGAAAGAGCCATTTATATCTGCAATCATGCATCTCCCATTGACATTTTCCTCATTATGTGGTTGACTCCGACCGG : 452
D-Gb2-LPAATB : GGAAATCCATTGAAGATCGAAGGAACGGAGTTCTCGAATGAAAGAGCCATTTATATCTGCAATCATGCATCTCCCATTGACATTTTCCTCATTATGTGGTTGACTCCGACCGG : 452
               GGAAATCCATTGAAGATCGAAGGAACGGAGTTCTCGAATGAAAGAGCCATTTATATCTGCAATCATGCATCTCCCATTGACATTTTCCTCATTATGTGGTTGACTCCGACCGG

                      460        *       480        *       500        *       520        *       540        *       560
A-Gh-LPAATB  : AACTGTCGGCATTGCAAAGAAAGAGGT------------------------------------AACGAATCATCTTTGGATTGATCGATCTGATCCAAGAGCTGCCATTAAGT : 529
A-Gb1-LPAATB : AACTGTCGGCATTGCAAAGAAAGAGATCATATGGTACCCCTTGTTCGGACAACTATATGTTTTAGCGAATCATCTTTGGATTGATCGATCTGATCCAAGAGCTGCCATTAAGT : 565
A-Gb2-LPAATB : AACTGTCGGCATTGCAAAGAAAGAGATCATATGGTACCCCTTGTTCGGACAACTATATGTTTTAGCGAATCATCTTTGGATTGATCGATCTGATCCAAGAGCTGCCATTAAGT : 565
D-Gh-LPAATB  : AACCGTCGGCATTGCAAAGAAAGAGGT------------------------------------AACGAATCATCTTCGGATTGATCGATCTGATCCAAGCACTGCCATTAAGT : 529
D-Gb1-LPAATB : AACCGTCGGCATTGCAAAGAAAGAGATCATATGGTACCCCTTGTTCGGACAACTATATGTTTTAGCGAATCATCTTCGGATTGATCGATCTGATCCAAGCACTGCCATTAAGT : 565
D-Gb2-LPAATB : AACCGTCGGCATTGCAAAGAAAGAGATCATATGGTACCCCTTGTTCGGACAACTATATGTTTTAGCGAATCATCTTCGGATTGATCGATCTGATCCAAGCACTGCCATTAAGT : 565
               AAC GTCGGCATTGCAAAGAAAGAG T                                    A CGAATCATCTT GGATTGATCGATCTGATCCAAG  CTGCCATTAAGT

                     *       580        *       600        *       620        *       640        *       660        *       6
A-Gh-LPAATB  : CCATGAAAAAGGCAGTACAGGCTGTTAAAAAACATGGTTTATCTCTGATTAATTTTTCCTGAGGGAACGAGGTCCAAAAATGGACGACTCCTTCCATTTAAAAAGGGTTTTGTT : 642
A-Gb1-LPAATB : CCATGAAAAAGGCAGTACAGGCTGTTAAAAAACATGGTTTATCTCTGATTAATTTTTCCTGAGGGAACGAGATCCAAAAATGGACGACTCCTTCCATTTAAAAAGGGTTTTGTT : 678
A-Gb2-LPAATB : CCATGAAAAAGGCAGTACAGGCTGTTAAAAAACATGGTTTATCTCTGATTAATTTTTCCTGAGGGAACGAGGTCCAAAAATGGACGACTCCTTCCATTTAAAAAGGGTTTTGTT : 678
D-Gh-LPAATB  : CCATGAAAAAGGCAGTTCAGGCTGTTAAAAAACATGGTTTATCTCTGATTGTTTTTCCTGAGGGAACGAGATCCAAAAATGGACGACTCCTTCCATTTAAAAAGGGTTTTGTT : 642
D-Gb1-LPAATB : CCATGAAAAAGGCAGTTCAGGCTGTTAAAAAACATGGTTTATCTCTGATTATTTTTCCTGAGGGAACGAGATCCAAAAATGGACGACTCCTTCCATTTAAAAAGGGTTTTGTT : 678
D-Gb2-LPAATB : CCATGAAAAAGGCAGTTCAGGCTGTTAAAAAACATGGTTTATCTCTGATTATTTTTCCTGAGGGAACGAGATCCAAAAATGGACGACTCCTTCCATTTAAAAAGGGTTTTGTT : 678
               CCATGAAAAAGGCAGT CAGGCTGTTAAAAAACATGGTTTATCTCTGATTaTTTTTCCTGAGGGAACGAG TCCAAAAATGGACGACTCCTTCCATTTAAAAAGGGTTTTGTT

               80        *       700        *       720        *       740        *       760        *       780        *
A-Gh-LPAATB  : CATTTAGCCATAGAGTCTGGCCTTCCCATAGTTCCAATAATCTTAACAGGCACTCATCTAGCATGGAGGAAAGGTAGCTTGCATGTTCGACCAGCACCAATATCCGTTAAGTA : 755
A-Gb1-LPAATB : CATTTAGCCATAGAGTCTGGCCTTCCCATAGTTCCAATAATCTTAACAGGCACTCATCTAGCATGGAGGAAAGGTAGCTTGCATGTTCGACCAGCACCAATATCCGTTAAGTA : 791
A-Gb2-LPAATB : CATTTAGCCATAGAGTCTGGCCTTCCCATAGTTCCAATAATCTTAACAGGCACTCATCTAGCATGGAGGAAAGGTAGCTTGCATGTTCGACCAGCACCAATATCCGTTAAGTA : 791
D-Gh-LPAATB  : CATTTAGCCATAGAGTCTGGCCTTCCCATAGTTCCAATAATCTTAACAGGCACTCATCTAGCATGGAGGAAAGGTAGCTTGCATGTTCGACCAGCACCAATATCCGTTAAGTA : 755
D-Gb1-LPAATB : CATTTAGCCATAGAGTCTGGCCTTCCCATAGTTCCAATAATCTTAACAGGCACTCATCTAGCATGGAGGAAAGGTAGCTTGCATGTTCGACCAGCACCAATATCCGTTAAGTA : 791
D-Gb2-LPAATB : CATTTAGCCATAGAGTCTGGCCTTCCCATAGTTCCAATAATCTTAACAGGCACTCATCTAGCATGGAGGAAAGGTAGCTTGCATGTTCGACCAGCACCAATATCCGTTAAGTA : 791
               CATTTAGCCATAGAGTCTGGCCTTCCCATAGTTCCAATAATCTTAACAGGCACTCATCTAGCATGGAGGAAAGGTAGCTTGCATGTTCGACCAGCACCAATATCCGTTAAGTA

                    800        *       820        *       840        *       860        *       880        *       900
A-Gh-LPAATB  : TCTTCCTCCAATTAAAACCGATAGTTGGACGGCTGACAAGATCAACGATTACATAAAAAATGGTGCATGACATATATGTTGAAAACCTTCCGGAGGCACAGAAACCGATTTCAA : 868
A-Gb1-LPAATB : TCTTCCTCCAATTAAAACCGATAGTTGGACGGCTGACAACATCAACGATTACATAAAAAATGGTGCATGACATATATGTTGAAAACCTTCCGGAGGCACAGAAACCGATTTCAA : 904
A-Gb2-LPAATB : TCTTCCTCCAATTAAAACCGATAGTTGGACGGCTGACAACATCAACGATTACATAAAAAATGGTGCATGACATATATGTTGAAAACCTTCCGGAGGCACAGAAACCGATTTCAA : 904
D-Gh-LPAATB  : TCTTCCTCCAATTAAAACCGATAGTTGGACGGCTGACAAGATCAACGATTACATAAAAAATGGTGCATGACATATATGTTGAAAACCTTCCGGAGGCACAGAAACCGATTTCAA : 868
D-Gb1-LPAATB : TCTTCCTCCAATTAAAACCGATAGTTGGACGGCTGACAACATCAACGATTACATAAAAAATGGTGCATGACATATATGTTGAAAACCTTCCGGAGGCACAGAAACCGATTTCAA : 904
D-Gb2-LPAATB : TCTTCCTCCAATTAAAACCGATAGTTGGACGGCTGACAACATCAACGATTACATAAAAAATGGTGCATGACATATATGTTGAAAACCTTCCGGAGGCACAGAAACCGATTTCAA : 904
               TCTTCCTCCAATTAAAACCGATAGTTGGACGGCTGACAA ATCAACGATTACATAAAAAATGGTGCATGACATATATGTTGAAAACCTTCCGGAGGCACAGAAACCGATTTCAA

                      *       920        *
A-Gh-LPAATB  : CCGATGACACCAAGAACAGTTCAAGATCATAA : 900
A-Gb1-LPAATB : CCGATGACACCAAGAACAGTTCAAGATCATAA : 936
A-Gb2-LPAATB : CCGATGACACCAAGAACAGTTCAAGATCATAA : 936
D-Gh-LPAATB  : CCGATGACACCAAGAACAGTTCAAGATCATAA : 900
D-Gb1-LPAATB : CCGATGACACCAAGAACAGTTCAAGATCATAA : 936
D-Gb2-LPAATB : CCGATGACACCAAGAACAGTTCAAGATCATAA : 936
               CCGATGACACCAAGAACAGTTCAAGATCATAA
```
